# Prevalence and risk factors for type 2 diabetes mellitus in women with gestational diabetes mellitus: a systematic review and meta-analysis

**DOI:** 10.3389/fendo.2024.1486861

**Published:** 2024-12-23

**Authors:** Kaiqi Chen, Lichao Tang, Xinwei Wang, Yunhua Li, Xijian Zhang, Shikui Cui, Wei Chen, Zhao Jin, Danping Zhu

**Affiliations:** ^1^ School of Basic Medical, Chengdu University of Traditional Chinese Medicine, Chengdu, China; ^2^ College of Education, Chengdu College of Arts and Sciences, Chengdu, China; ^3^ Department of Endocrinology, Chongqing Traditional Chinese Medicine Hospital, Chongqing, China; ^4^ Department of Pharmacy, Emergency General Hospital, Beijing, China

**Keywords:** diabetes, epidemiology, endocrinology, prenatal care, high-risk pregnancy, gestational diabetes mellitus, women’s health

## Abstract

**Introduction:**

This study aims to explore the risk factors in the progression of gestational diabetes mellitus (GDM) to type 2 diabetes mellitus (T2DM).

**Material and methods:**

Relevant studies were comprehensively searched from PubMed, Web of Science, Cochrane Library, and Embase up to March 12. Data extraction was performed. Differences in risk factors were presented as odds ratios (OR) and corresponding 95% confidence intervals (CI). The quality of the included studies was assessed through the Newcastle-Ottawa Scale and the Agency for Healthcare Research and Quality scale.

**Results:**

This meta-analysis encompassed 46 studies involving a total of 196,494 patients. The factors most strongly associated with the risk of developing T2DM following GDM were the use of progestin-only contraceptives (odds ratio [OR]: 2.12, 95% confidence interval [CI] = 1.00–4.45, P = 0.049), recurrence of GDM (OR: 2.63, 95% CI = 1.88–3.69, P < 0.001), insulin use during pregnancy (OR: 4.35, 95% CI = 3.17–5.96, P < 0.001), pre-pregnancy body mass index (BMI) (OR: 2.97, 95% CI = 2.16–4.07, P < 0.001), BMI after delivery (OR: 4.17, 95% CI = 2.58–6.74, P < 0.001), macrosomia (OR: 3.30, 95% CI = 1.45–7.49, P = 0.04), hypertension (OR: 5.19, 95% CI = 1.31–20.51, P = 0.019), and HbA1c levels (OR: 3.32, 95% CI = 1.81–6.11, P < 0.001). Additionally, age (OR: 1.71, 95% CI = 1.23–2.38, P = 0.001), family history of diabetes (OR: 1.47, 95% CI = 1.27–1.70, P < 0.001), BMI during pregnancy (OR: 1.06, 95% CI = 1.00–1.12, P = 0.056), fasting blood glucose (FBG) (OR: 1.58, 95% CI = 1.36–1.84, P < 0.001), 1-hour oral glucose tolerance test (OGTT) (OR: 1.38, 95% CI = 1.02–1.87, P = 0.037), and 2-hour OGTT (OR: 1.54, 95% CI = 1.28–1.58, P < 0.001) were identified as moderate-risk factors for the development of T2DM.

**Conclusion:**

The systematic review and meta-analysis identified several moderate- to high-risk factors associated with the progression of T2DM in individuals with a history of GDM. These risk factors include the use of progestin-only contraceptives, pre-pregnancy BMI, BMI after delivery, macrosomia, hypertension, persistently elevated levels of HbA1c, fasting blood glucose (FBG), 1-hour and 2-hour oral glucose tolerance tests (OGTT), age, and family history of diabetes. Our findings serve as evidence for the early prevention and clinical intervention of the progression from GDM to T2DM and offer valuable insights to guide healthcare professionals in formulating customized management and treatment strategies for female patients with diverse forms of GDM.

**Systematic review registration:**

https://www.crd.york.ac.uk/PROSPERO/, identifier CRD42024545200.

## Highlights

- This meta-analysis identified multiple moderate- to high-risk factors for T2DM in GDM patients: progestin-only contraceptives, BMI, macrosomia, hypertension, and HbA1c levels, among others, and provided substantial evidence to inform early preventive measures and clinical interventions.

## 1 Introduction

Gestational diabetes mellitus (GDM) is defined as a state of hyperglycemia during pregnancy that resolves after delivery in women who have never been diagnosed with diabetes. This condition involves glucose intolerance. It was conceptualized by Carrington in 1957 (1) and gained widespread recognition in the 1960s. Due to the lack of standardized diagnostic criteria, the prevalence of GDM ranges from 1% to over 30% globally (2). The median prevalence is the highest in the Middle East and North Africa (15.2%), and the lowest (6.1%) is in Europe. Although earlier studies posited GDM as a benign condition (3), recent evidence suggests that it heightens the risk of various complications like macrosomia and preeclampsia for both babies and mothers during pregnancy. GDM may lead to poor pregnancy outcomes (4) and have long-term effects on the health of mothers and children, such as elevating the risk of obesity and premature cardiovascular disease (5, 6).

Due to changes in maternal demographics and rising global obesity rates in recent years, there is a significant risk of the progression from GDM to type 2 diabetes mellitus (T2DM), which poses a pressing threat to public health. Plenty of studies have indicated a 6-10 times risk of progressing to T2DM in women with GDM in contrast to the general pregnant population (7–10). A 2016 global review has estimated that the cumulative probability of developing T2DM after GDM ranges from 2% to 66%, with substantial regional variations; however, the risk remains significantly higher in women with GDM than in the general female population (11). A 2020 meta-analysis, encompassing 129 studies, reported a relative risk (RR) of 8.3 for the development of T2DM following GDM, with nearly 17% of women with GDM progressing to T2DM (12).

Despite the proven correlation between GDM and T2DM, there is no comprehensive analysis of relevant risk factors for the progression from GDM to T2DM. A meta-analysis and systematic review of risk factors are necessary. Therefore, this study aims to thoroughly evaluate the risk of T2DM among women with GDM through a meta-analysis, and offer evidence-based references for clinicians in the development of postpartum screening plans and intervention strategies during pregnancy, thereby improving T2DM prevention and intervention in female GDM patients.

## 2 Materials and methods

Our research adhered to the Preferred Reporting Items for Systematic Reviews and Meta-Analyses 2020 (PRISMA 2020) Declaration (13). It has been registered with PROSPERO under registration number CRD42024545200.

### 2.1 Search strategy

A thorough and systematic search was carried out across PubMed, EMBASE, Cochrane Library, and Web of Science up to March 12, 2024. Medical subject headings (MeSH) and free-text terms were used for the search. The keywords were: “diabetes mellitus, type 2”, “diabetes, gestational”, “ketosis resistant diabetes mellitus”, “non-insulin dependent diabetes”, “stable diabetes mellitus”, “MODY”, “diabetes mellitus”, “maturity onset diabetes”, “type 2 diabetes”, “adult onset diabetes”, “diabetes type ii”, “insulin independent diabetes”, “DM 2”, “T2DM”, and “pregnancy diabetes”. The details are presented in **Supplementary Table S1**.

### 2.2 Inclusion criteria

1. The study population comprised women who were diagnosed with GDM by a physician, aged 18 or more, and subsequently developed T2DM.
2. The original study employed multivariate logistic regression to pinpoint one or more T2DM-associated risk factors, including demographic and lifestyle characteristics (e.g., age, family history of diabetes), pregnancy-related factors (e.g., insulin use during pregnancy, inter-pregnancy intervals), and laboratory analyses (e.g., FBG, OGTT).
3. The studies are prospective or retrospective cohort studies, cross-sectional or case-control studies.
4. The research offered data such as odds ratios (OR), RR, hazard ratios (HR) with 95% CI, or sufficient data for calculation.
5. Studies were published in English.

### 2.3 Exclusion criteria

1. Patients who were not diagnosed with GDM were excluded.
2. Duplicates, animal studies, reviews, letters, conference abstracts, case reports, case series, and editorials were removed.
3. Studies were excluded if they did not report endpoints related to risk factors or if the full text could not be accessed.

### 2.4 Data extraction and quality assessment

Data extraction was independently completed by two experienced reviewers. Discrepancies were addressed through discussion or consultation with a third reviewer. A standardized Microsoft Excel spreadsheet, provided by Cochrane, was used for data collection. The extracted information included the first author, publication year, country or region, study design, total sample size, prevalence of T2DM, diagnostic criteria for GDM and T2DM, and other pertinent factors. Furthermore, risk factors for T2DM identified after multifactorial logistic regression analysis, such as OR and 95% CI, were extracted. The quality and methodological rigor of all selected studies were evaluated through the Newcastle-Ottawa Scale (NOS) (14) for cohort and case-control studies, and through the Agency for Healthcare Research and Quality (AHRQ) (15) guidelines for cross-sectional studies. The NOS includes two primary components: one for cohort studies and one for case-control studies, each of which has three domains: selection, comparability, and outcome or exposure. The maximum score for each domain is nine points. The scores are categorized into three levels: low (four points or fewer), moderate (five or six points), and high (seven points or more).

### 2.5 Statistical analyses

The meta-analysis was performed with the help of STATA. The risk factors for T2DM were analyzed via OR values and their associated 95% CI. A fixed-effects model (FEM) was constructed for the meta-analysis when statistical heterogeneity was minimal (P > 0.10 and I² ≤ 50%); otherwise, a random-effects model (REM) was employed. In the case of observed heterogeneity, the results of the fixed-effects model and the random-effects model were compared (16). If significant discrepancies were identified, a sensitivity analysis was performed by systematically excluding each study to explore potential sources of heterogeneity. Moreover, subgroup analyses were performed to further elucidate the sources of heterogeneity. The primary outcome of this study was the risk factors for T2DM among patients with GDM. Subgroup analyses based on geographic location, study design, and other relevant variables were carried out to enhance the robustness of our findings. Risk factors demonstrating statistical significance were classified as high risk (OR≥2), moderate risk (1< OR < 2), or protective factor(OR< 1). Publication bias was assessed via funnel plots and Egger’s test. A p-value of less than 0.05 signifies statistical significance.

## 3 Results

### 3.1 Search results and study characteristics

**Figure 1:**
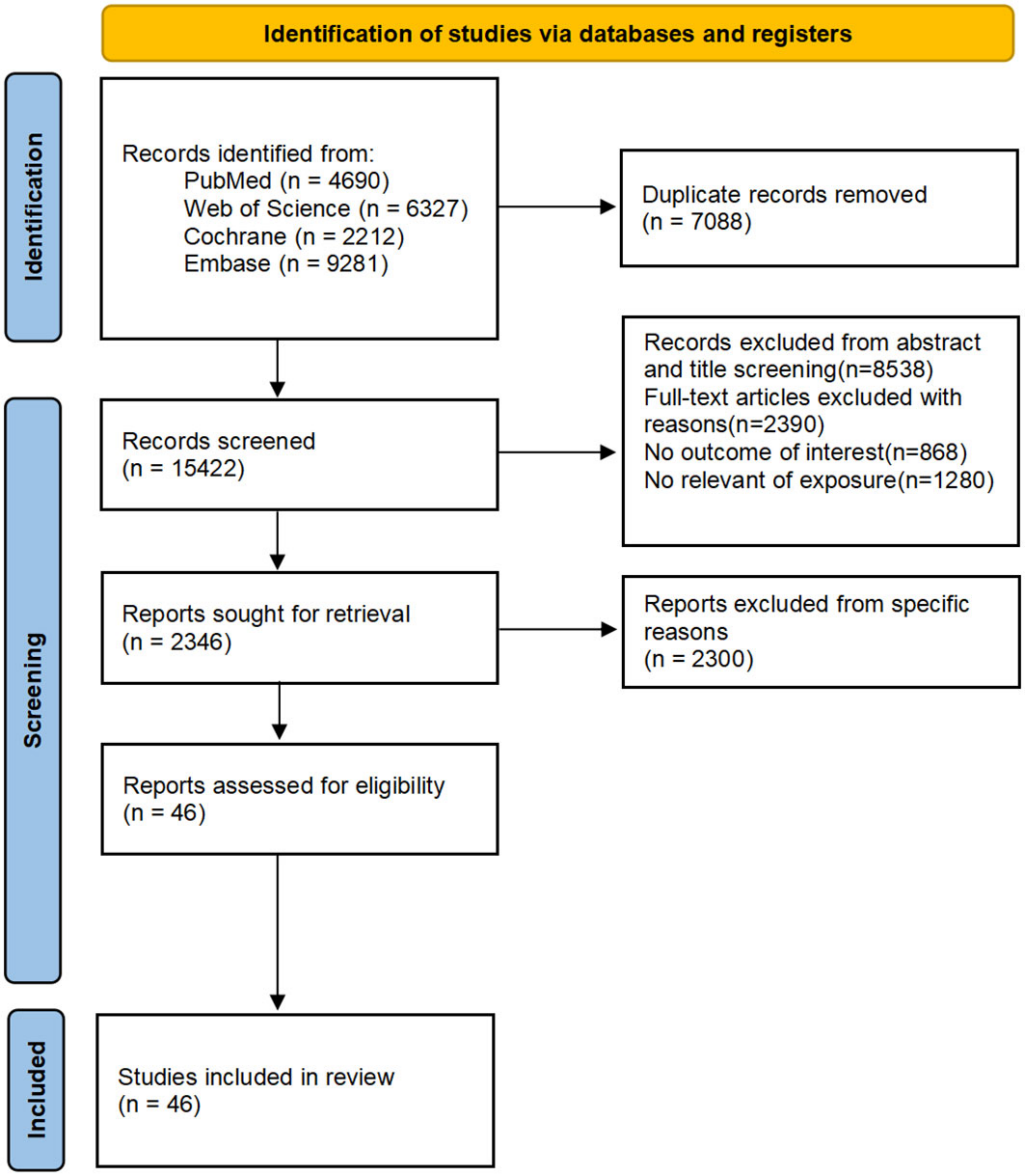
Flow chart of study selection.

Our initial search yielded 22,510 articles, with 4,690 from PubMed, 6,327 from Web of Science, 9,281 from Embase, and 2,212 from Cochrane. After the exclusion of duplicates and irrelevant studies based on titles and abstracts, the full texts of the remaining 2,346 studies were reviewed. Ultimately, 46 studies were selected for inclusion. The study selection process is outlined in **Figure 1**.

**Table 1** presents the characteristics of the included articles, along with an assessment of the quality of each study. The studies were published between 1997 and 2024. Among the studies, six (17–22) were in Europe, 15 in Asia (23–36), 21 in North America (18, 37–56), four in Oceania (57–60), and one in Africa (61). Of the ultimately selected 46 studies, 58 distinct risk factors were identified. Demographic and lifestyle factors included: age, family history of diabetes, use of progestin-only contraceptives, breastfeeding practices, higher education, ethnicity (Asian, White, Hispanic, African American), healthy dietary patterns, physical activity and sedentary behaviors, habitual iron intake, low-carbohydrate diet scores, dietary intakes, habitual alcohol consumption, and habitual coffee consumption. Pregnancy-related factors encompassed: early diagnosis of GDM, recurrence of GDM, insulin use during pregnancy, pre-pregnancy BMI, BMI during pregnancy, BMI postpartum, weight change, gestational interval, parity, waist circumference, macrosomia, neonatal birth weight, hypertension, spontaneous abortions, skin fold thickness measurements (suprailiac, tricep, subscapular), waist/hip ratio, preeclampsia, sex of the baby, and exposure to particulate matter (PM). Laboratory indicators included: HbA1c, FBG, 1-hour and 2-hour OGTT values, elevated homocysteine levels, positive autoantibodies, neonatal hypoglycemia, basal glucose clearance, OGTT total area, 30-minute insulin response during OGTT, high-density lipoprotein cholesterol, triglycerides, uric acid, circulating concentrations of branched-chain amino acids, sex hormone-binding globulin (SHBG), alanine aminotransferase (ALT), metabolomics score, and amino acid and lipid sub-scores.

**Table 1:** Characteristics of studies.

Author	Region	Study design	Sample size	T2DM	GDM criteria	T2DM criteria	Risk factors
JONATHAN R. STEINHART-1997	America	Retrospective	111	47	NA	WHO	FBG; spontaneous abortions; GTT total; recurrent GDM; insulin use
Siri L-1998	America	Retrospective	904	169	NDDG	NDDG	Contraception
NAM H. CHO1-2005	Korea	Prospective	170	18	NDDG	WHO	Age; Gestational age at diagnosis of GDM; Pre-pregnancy BMI; Positive family history of diabetes; Higher FBG; Higher homocysteine level
N. Wah Cheung-2005	Australia	Retrospective	102	30	NA	NA	BMI; FBG; OGTT 2-h; Insulin use in pregnancy
NAM H. CHO2-2005	Korea	Prospective	909	116	NDDG	NDDG	Suprailiac skin fold thickness; tricep skin fold thickness; waist/hip ratio; BMI; subscapular skin fold thickness; weight; waist
Anny H Xiang-2006	America	Prospective	526	106	NA	ADA	Depo-medroxyprogesterone acetate
Kristian Lo¨bner-2006	Germany	Prospective	302	130	German Diabetes Association	ADA	Autoantibody positive; Insulin in pregnancy; BMI; Previous pregnancies
Anna J Lee-2007	Australia	Retrospective	5,470	405	ADPSG	WHO, 1998	Race; height; age; parity; BMI; birth weight; BwtGC; gestational age; insulin use in pregnancy; family history of diabetes; FBG; 1-h blood glucose; 2-h blood glucose
C Russell-2007	Canada	Retrospective	1,401	251	Canadian Diabetes Association	NA	Weight; insulin use; neonatal hypoglycemia; subsequent pregnancies GDM
Anny H. Xiang-2010	Spain	Prospective	72	31	NA	ADA	Intravenous glucose tolerance tests; basal glucose clearance; OGTT total area; OGTT-30 insulin; weight change; additional pregnancy; progestin-only method
Christian S. Go¨bl-2011	Austria	Prospective	110	23	IADPSG	WHO	OGTT; Age; HDL-C; Insulin during pregnancy; RRS/RRD; TG; BMI; WC; FPG
A. H. Xiang-2011	America	Retrospective	12,998	1539	IADPSG	ADA	Race
Tobias DK-2012	America	Prospective	4,413	491	NA	NDDG	Healthful dietary patterns
Yujie Wang-2012	America	Prospective	1,142	327	ADA	WHO	Age; BMI; race
Denice S. Feig-2013	Canada	Retrospective	3,576	1292	NA	NA	Preeclampsia; gestational hypertension
Bao W-2014	America	Prospective	4,554	635	NA	NDDG	Physical activity and sedentary behaviors
Huikun Liu-2014	China	Retrospective	1,263	83	WHO	ADA	BMI
R.Retnakaran-2015	Canada	Retrospective	23,363	5483	NA	NA	Pathophysiology; sex of the baby
Claire E Eades-2015	Scotland	Prospective	164	41	an FBG of over 5.5 mmol/l-1 or blood glucose reading two hours (2 h BG) after an OGTT of over 9 mmol/l-1	WHO	Weight gain during pregnancy; use of insulin during pregnancy; HbA1c levels at diagnosis of GDM; FBG
Valizadeh M-2015	Iran	Retrospective	110	36	NA	NA	Parity; delivery and follow-up lab test interval; FBG; maternal weight; BMI; waist circumference; neonatal birth weight; age; family history of diabetes mellitus; history of delivering macrosomic neonate; insulin use
Joon Ho Moon-2015	Korea	Prospective	418	53	NDDG	ADA	Postpartum BMI change
Pei-Chao LIN-2015	China	Retrospective	71	29	NDDG	ADA	BMI; Insulin use during pregnancy
Piotr Molęda-2016	Poland	Retrospective	199	13	OGTTs	WHO	Uric acid
Catherine R Chamberlain-2016	Australia	Retrospective	289	82	ADPSG	WHO	BMI; breastfeeding;
Bao W1-2016	America	Prospective	3,976	641	NA	ADA	Habitual iron intake
Bao W2-2016	America	Prospective	1,695	259	NA	ADA	BMI; weight change
Bao W3-2016	America	Prospective	4,502	722	NA	ADA	Low-carbohydrate diets scores
Deirdre K-2018	America	Retrospective	347	172	NA	NA	Dietary Intakes; circulating concentrations of branched-chain amino acids
Casagrande SS-2018	America	Prospective	568	112	NA	NA	Age; years since GDM diagnosis; family history of diabetes; BMI; education
Yukari Kugishima-2018	Japan	Retrospective	306	32	IADPSG	WHO	BMI; 2-h PG; HbA1c; Insulin therapy during pregnancy
Judith Bernstein-2019	Boston	Retrospective	1,091	58	NA	NA	GDM recurrence; delivery interval
Tawanda Chivese-2019	South Africa	Retrospective	150	47	IADPSG	WHO, 2006	Waist circumference; Hip circumference; BMI; age at follow-up; secondary and matric education; dyslipidemia; hypertension; family history of diabetes; total physical activity
Ley SH-2020	America	Retrospective	4,372	873	NA	NDDG	Lactation duration
Kawasaki M-2020	East Asia	Retrospective	399	43	Japan Society of Obstetrics and Gynecology criteria; IADPSG criteria	WHO	BMI; ppOGTT 2-h plasma glucose; ppOGTT HbA1c ≥5.7% age at childbirth; family history of diabetes; GDM diagnosis before 20 weeks gestation; use of insulin during pregnancy; macrosomia
Dayeon Shin-2021	Korea	Prospective	629	NA	NA	T2DM refers to a woman diagnosed with diabetes by a doctor or an FBG level ≥126 mg/dL.	Pre-pregnancy BMI
Pandora L. Wander-2021	America	Prospective	335	/	NA	ADA	Adiposity and related biomarkers; sex hormone-binding globulin; alanine aminotransferase
Stefanie N Hinkle-2021	America	Retrospective	4,740	897	NA	NDDG	Habitual alcohol consumption
Chiou YL-2021	China	Prospective	57	24	NA	HbA1c ≥ 6.5%	Education level; pre-pregnancy BMI; 100-g OGTT FBG; 100-g OGTT 1-h blood glucose; 75-g OGTT FBG; 75-g OGTT 2-h blood glucose
Anna J Wood-2021	Australia	Prospective	82	11	IADPSG; WHO	WHO	Demographics; age; multiparity; family history of diabetes; increased glucose values; insulin use; BMI
Enav Yefet-2022	Israel	Retrospective	788	178	NA	NDDG	Recurrent GDM; maternal and obstetrical characteristics of the GDM pregnancy; the consecutive pregnancy including BMI gain and inter-pregnancy interval
Jiaxi Yang-2022	America	Prospective	4,522	979	NDDG	ADA	Habitual coffee consumption
Mi Jin Choi-2022	Korea	Retrospective	5,781	302	ADA	WHO	BMI; FBG; age; family history of diabetes; hypertension; insulin use during pregnancy
Roosa P-2022	Finland	Prospective	96,353	5370	IADPSG	NA	Insulin therapy during GDM
Yumei Wei-2022	China	Retrospective	1,002	23	IADPSG	ADA	Pre-pregnancy BMI; age; IFG; history of macrosomia; weight change between twice pregnancy; gestational interval
Amir Naeh	Israel	Retrospective	1,812	119	NDDG	WHO	Multifetal pregnancy
Deirdre K Tobias-2024	America	Prospective	350	175	NA	ADA	Metabolomics score; amino acid and lipid sub‐scores

GTT, Glucose Tolerance Test; GDM, Gestational Diabetes Mellitus; BMI, Body Mass Index; FBG, Fasting Blood Glucose; OGTT, Oral Glucose Tolerance Test; BwtGC, Birth Weight Gestational Centile; HDL-C, High-Density Lipoprotein Cholesterol; RRS/RRD, Systolic and Diastolic Blood Pressure; TG, Triglycerides; WC, Waist Circumference; FPG, Fasting Plasma Glucose; PG, Plasma Glucose; PP, Postpartum; PM, Particulate Matter. NA, Not Applicable.

Due to the absence of sufficient studies, a meta-analysis on 31 risk factors was impossible. Hence, only 26 (17–20, 22–35, 37–40, 42–44, 50–52, 57–61) of the 58 identified risk factors were meta-analyzed. The results of this analysis, as well as those from original outcome studies, are presented in **Table 2**. We examined the impact of these 26 risk factors on the incidence of T2DM in women with GDM. Notably, publication bias was detected for several factors, including insulin use during pregnancy, hypertension, and the 2-hour OGTT. Detailed information on publication bias can be found in **Supplementary Table S2**.

**Table 2:** Categorical analysis on the correlation between risk factors for T2DM and GDM.

Risk factors	No. of studies	Heterogeneity		Effective models	OR (95% CI)	Z	*p*
		I² (%)	*p*				
Demographic and lifestyle characteristics							
Age	12	95.9	0.000	Random	1.71 (1.23, 2.38)	3.18	0.001
Family history of diabetes	9	42.4	0.085	Random	1.60 (1.26, 2.04)	3.8	<0.001
Use of progestin-only contraceptive	3	61	0.077	Random	2.12 (1.00, 4.45)	1.97	0.049
Breastfeeding	2	0.0	0.87	Fixed	0.81 (0.39, 1.68)	0.56	0.573
Greater education	4	62.7	0.045	Random	0.53 (0.20, 1.37)	1.32	0.188
Asian	3	0.0	0.538	Fixed	6.22 (4,97, 7.79)	15.91	<0.001
White	2	75.5	0.043	Random	5.52 (3.96, 7.69)	10.08	<0.001
Hispanic	2	0.0	0.434	Fixed	7.75 (6.86, 8.76)	32.84	<0.001
African American	2	67.4	0.080	Random	8.38 (6.35, 11.05)	15.02	<0.001
Pregnancy-related factors							
Weight change	3	65	0.057	Random	1.03 (0.90, 1.19)	0.46	0.647
Parity	2	56.5	0.129	Random	1.12 (0.84, 1.49)	0.79	0.432
Waist circumference	4	86.8	0.000	Random	1.12 (0.98, 1.29)	1.68	0.094
Macrosomia	3	0.0	0.671	Fixed	3.30 (1.45, 7.49)	2.85	0.004
Neonatal birth weight	3	74.3	0.049	Random	1.22 (0.74, 2.00)	0.77	0.442
Insulin use in pregnancy	14	82.6	0.000	Random	4.35 (3.17, 5.96)	9.13	<0.001
Early diagnosis GDM	5	46.1	0.115	Fixed	0.96 (0.93, 0.99)	1.13	0.26
GDM recurrence	4	0.0	0.476	Fixed	2.63 (1.88, 3.69)	5.62	<0.001
Hypertension	4	96.6	0.000	Random	5.19 (1.31, 20.51)	2.35	0.019
Gestational interval	2	0.0	0.606	Fixed	1.68 (1.09, 2.58)	2.37	0.018
Pre-pregnancy BMI	15	93.8	0.000	Random	2.93 (2.11, 4.07)	6.44	<0.001
BMI in pregnancy	5	70.1	0.010	Random	1.26 (0.99, 1.60)	1.91	0.056
BMI after delivery	9	99.5	0.000	Random	4.58 (2.87, 7.30)	6.38	<0.001
Laboratory indicators							
HbA1c	3	0.0	0.609	Fixed	3.32 (1.81, 6.11)	3.86	<0.001
FBG	12	96	0.000	Random	1.58 (1.36, 1.84)	5.91	<0.001
OGTT 1-h	4	99.5	0.000	Random	1.38 (1.02, 1.87)	2.08	0.037
OGTT 2-h	8	80	0.000	Random	1.54 (1.28, 1.85)	4.62	<0.001

### 3.2 Risk of bias assessment

The quality of the 42 included cohort studies was assessed through NOS, with scores ranging from five to eight stars, indicating a relatively low risk of bias. A summary of the quality assessment is presented in **Supplementary Table S3**. The four cross-sectional studies were evaluated using the AHRQ assessment tool, and their results also indicated reliable quality. More details are provided in **Supplementary Table S3**.

### 3.3 Meta-analysis

#### 3.3.1 Demographic and lifestyle characteristics

A meta-analysis was performed to examine the influence of age (19, 20, 23, 26, 30, 34, 35, 42, 50, 58, 60), family history of diabetes (20, 23, 26, 30, 34, 50, 58, 60, 61), use of progestin-only contraceptives (18, 38, 39), breastfeeding (59, 60), higher educational attainment (32, 50, 60, 61), and race [Asian (18, 42, 58), White (18, 42), Hispanic (18, 42) African American (18, 42)] on the progression to T2DM from GDM. Of particular note, the use of progestin-only contraceptives (OR: 2.12, 95% CI: 1.00-4.45, P=0.049) was identified to be a high-risk factor. Age (OR: 1.71, 95% CI: 1.23-2.38, P=0.001) and a family history of diabetes (OR: 1.47, 95% CI: 1.27-1.70, P<0.001) were deemed moderate-risk factors for T2DM. Breastfeeding (OR: 0.81, 95% CI: 0.39-1.68, P=0.573) and higher educational attainment (OR: 0.53, 95% CI: 0.20-1.37, P=0.188) were considered protective factors against T2DM, though the results did not reach statistical significance. In terms of race, all four groups had an elevated risk for progression from GDM to T2DM; White individuals had a relatively lower risk (OR: 5.52, 95% CI: 3.96-7.69, P<0.001) and African Americans had a relatively higher risk (OR: 8.38, 95% CI: 6.35-11.05, P<0.001).

Significant heterogeneity was observed among the studies in terms of age, use of progestin-only contraceptives, higher educational attainment, and race (White and African American), with I² values of 95.9%, 61%, 62.7%, 75.5%, and 67.4%, respectively. However, a comparison of results through both fixed-effect and random-effect models (61) revealed no significant differences for the remaining factors, excluding education and the use of progestin-only contraceptives. This suggests that the results were stable. To further assess this, sensitivity analyses were conducted by sequentially excluding studies that considered educational attainment and progestin-only contraceptives as risk factors. The analysis revealed that the study by Casagrande SS-2018 had a notable influence on the heterogeneity observed for educational attainment. Many of the other studies included were retrospective, which could have contributed to the observed heterogeneity. However, the source of heterogeneity in studies involving progestin-only contraceptives remained unclear. Subgroup analysis based on region, study design, sample size, and diagnostic criteria for T2DM and GDM were subsequently conducted to find potential sources of heterogeneity (**Tables 3**, **4**). No significant publication bias was noted for relevant factors (P > 0.05).

**Table 3:** Subgroup analysis of incidence for the development of T2DM in GDM women.

Subgroup	No	Incidence of GDM (95% CI)	heterogeneity		Effective model	Z	*p*
			I² (%)	*p*			
Location							
North America	20	0.22 (0.19, 0.25)	99.2	0.000	Random	14.01	<0.001
Asia	12	0.14 (0.11, 0.18)	97.3	0.000	Random	8.49	<0.001
Oceania	5	0.19 (0.08, 0.30)	96.0	0.000	Random	3.44	0.001
Europe	5	0.24 (0.11, 0.37)	98.3	0.000	Random	3.58	<0.001
Study design							
Retrospective	23	0.20 (0.16, 0.24)	99.5	0.000	Random	10.22	<0.001
Prospective	20	0.21 (0.18, 0.25)	99.3	0.000	Random	11.40	<0.001
Sample							
<500	19	0.28 (0.21, 0.35)	96.8	0.000	Random	7.96	<0.001
>500	25	0.16 (0.13, 0.19)	99.7	0.000	Random	10.17	<0.001
Diagnostic criteria of GDM							
ADA	2	0.17 (-0.07, 0.40)	99.7	0.000	Random	1.41	0.157
IADPSG	8	0.12 (0.08, 0.15)	98.8	0.000	Random	7.06	<0.001
ADPSG	2	0.17 (-0.03, 0.38)	98.4	0.000	Random	1.65	0.099
NDDG	6	0.18 (0.13, 0.23)	94.5	0.000	Random	7.43	<0.001
Others	3	0.29 (0.13, 0.44)	97.1	0.000	Random	3.56	<0.001
Diagnostic criteria of T2DM							
WHO	13	0.17 (0.14, 0.21)	97.7	0.000	Random	9.25	<0.001
NDDG	7	0.17 (0.14, 0.20)	97.4	0.000	Random	10.37	<0.001
ADA	13	0.21 (0.17, 0.26)	99.1	0.000	Random	9.94	<0.001

**Table 4:** Subgroup analysis of risk factors for the development of T2DM in GDM women.

Risk factors	No. of studies	Heterogeneity		OR (95% CI)	*p*
		I² (%)	*p*		
***Age***	12	95.9	0.000	1.71 (1.23, 2.38)	0.001
Location					
North America	2	0	0.882	5.28 (4.29, 6.51)	<0.001
Asia	5	45.2	0.121	1.31 (1.09, 1.58)	0.091
Oceania	2	84.5	0.011	1.27 (0.79, 2.05)	0.329
Europe	2	0	0.717	2.78 (1.43, 5.39)	0.002
Africa	1	-	-	0.90 (0.80, 1.01)	0.064
Sample					
<500	7	76	0.000	1.43 (0.94, 2.18)	0.092
>500	5	98.4	0.000	2.17 (1.02, 4.61)	0.045
Study design					
Retrospective	6	71.9	0.003	1.07 (0.92, 1.24)	0.361
Prospective	6	82.8	0.000	3.01 (1.65, 5.46)	<0.001
Diagnostic criteria of GDM					
ADA	2	95.3	0.000	3.04 (1.00, 9.22)	0.049
IADPSG	3	57.7	0.094	1.61 (1.11, 2.33)	0.011
NDDG	1	-	-	2.03 (0.68, 6.04)	0.203
Others	4	69.5	0.02	0.97 (0.83, 1.12)	0.665
Diagnostic criteria of T2DM					
WHO	9	96.9	0.000	1.66 (1.12, 2.46)	0.011
ADA	1	-	-	1.28 (1.01, 1.62)	0.038
***Insulin use in pregnancy***	14	82.6	0.000	4.35 (3.17, 5.96)	<0.001
Location					
North America	2	0	0.623	3.81 (2.11, 6.88)	<0.001
Asia	5	79.8	0.000	4.44 (1.86, 10.56)	0.001
Oceania	4	71.9	0.014	5.34 (2.63, 10.83)	<0.001
Europe	3	0	0.424	3.82 (3.58, 4.08)	<0.001
Sample					
<500	10	24.9	0.215	3.54 (2.77, 4.53)	<0.001
>500	4	95.1	0.000	6.08 (3.36, 10.99)	<0.001
Study design					
Retrospective	9	78.5	0.000	4.52 (2.76, 7.42)	<0.001
Prospective	5	0	0.597	3.82 (3.59, 4.08)	<0.001
Diagnostic criteria of GDM					
ADA	1	-	-	9.83 (5.78, 16.74)	<0.001
IADPSG	3	0	0.447	3.36 (1.73, 6.54)	<0.001
NDDG	1	-	-	19.66 (4.00, 96.66)	<0.001
Others	7	89.6	0.000	4.03 (2.71, 5.98)	<0.001
Diagnostic criteria of T2DM					
WHO	8	79.3	0.000	4.30 (2.47, 7.48)	<0.001
ADA	2	65.7	0.088	7.74 (2.03, 29.48)	0.003
***FBG***	12	96	0.000	1.58 (1.36, 1.84)	<0.001
Location					
North America	1	-	-	11.05 (1.65, 74.09)	0.013
Asia	6	95.5	0.000	2.28 (1.11, 4.68)	0.024
Oceania	4	81	0.001	1.59 (1.20, 2.11)	0.001
Europe	1	-	-	3.94 (0.92, 16.89)	0.065
Sample					
<500	9	79.3	0.000	2.34 (1.47, 3.70)	<0.001
>500	3	99.1	0.000	1.46 (1.21, 1.77)	<0.001
Study design					
Retrospective	6	98	0.000	1,57 (1.32, 1.87)	<0.001
Prospective	6	76	0.001	1.93 (1.10, 3.40)	0.022
Diagnostic criteria of GDM					
ADA	1	-	-	4.89 (3.51, 6.81)	<0.001
IADPSG	2	36.7	0.209	2.54 (1.61, 4.02)	0.002
NDDG	1	-	-	4.00 (1.41, 11.41)	0.009
Others	3	71.2	0.031	1.36 (1.09, 1.71)	0.007
Diagnostic criteria of T2DM					
WHO	9	91.6	0.000	2.27 (1.37, 3.78)	0.002
NDDG	1	-	-	1.03 (1.02, 1.05)	<0.001
***Hypertension***	4	96.6	0.000	5.19 (1.31, 20.51)	0.019
Location					
North America	1	-	-	18.49 (17.12, 19.96)	<0.001
Asia	1	-	-	2.21 (1.34, 3.65)	0.002
Oceania	1	-	-	3.29 (1.41, 7.68)	0.006
Africa	1	-	-	5.00 (1.60, 15.61)	0.006
Sample					
<500	2	0	0.563	3.82 (1.93, 7.54)	<0.001
>500	2	98.5	0.000	6.49 (0.81, 52.02)	0.078
Study design					
Retrospective	3	97.2	0.000	6.00 (1.17, 30.85)	0.032
Prospective	1	-	-	3.29 (1.41, 7.68)	0.006
Diagnostic criteria of GDM					
ADA	1	-	-	2.21 (1.34, 3.65)	0.002
IADPSG	1	-	-	3.29 (1.41, 7.68)	0.006
Others	1	-	-	5.00 (1.60, 15.61)	0.006
Diagnostic criteria of T2DM					
WHO	3	0	0.378	2.68 (1.79, 4.01)	<0.001
***OGTT 1-h***	4	99.5	0.000	1.38 (1.02, 1.87)	0.037
Location					
Asia	2	75.2	0.045	1.12 (0.87, 1.44)	0.38
Oceania	2	41.3	0.192	1.53 (1.48, 1.58)	<0.001
Sample					
<500	2	65.9	0.087	1.57 (1.06, 2.33)	0.024
>500	2	99.8	0.000	1.24 (0.83, 1.86)	0.289
Study design					
Retrospective	2	99.8	0.000	1.24 (0.83, 1.86)	0.289
Prospective	2	65.9	0.087	1.57 (1.06, 2.33)	0.024
Diagnostic criteria of GDM					
IADPSG	1	-	-	1.98 (1.35, 2.91)	0.001
Others	1	-	-	1.53 (1.48, 1.58)	<0.001
Diagnostic criteria of T2DM					
WHO	3	32.7	0.227	1.53 (1.48, 1.58)	<0.001
NDDG	1	-	-	1.01 (1.01, 1.02)	<0.001
***Waist circumference***	4	86.8	0.000	1.12 (0.98, 1.29)	0.094
Location					
Asia	2	91.5	0.001	1.88 (0.51, 6.88)	0.343
Oceania	1	-	-	3.97 (1.34, 11.80)	0.013
Africa	1	-	-	1.10 (1.05, 1.15)	<0.001
Sample					
<500	3	82.5	0.003	1.07 (0.97, 1.18)	0.181
>500	1	-	-	3.86 (1.81, 8.24)	<0.001
Study design					
Retrospective	2	82.6	0.017	1.06 (0.98, 1.14)	0.136
Prospective	2	0	0.967	3.90 (2.09, 7.26)	<0.001
Diagnostic criteria of GDM					
IADPSG	1	-	-	3.97 (1.34, 11.80)	0.013
NDDG	1	-	-	3.86 (1.81, 8.24)	<0.001
Others	1	-	-	1.10 (1.05, 1.15)	<0.001
Diagnostic criteria of T2DM					
WHO	2	81.2	0.021	1.85 (0.54, 6.38)	0.328
NDDG	1	-	-	3.86 (1.81, 8.24)	<0.001
***Early diagnosis GDM***	5	46.1	0.115	0.96 (0.93, 0.99)	0.26
Location					
Asia	3	72.8	0.025	1.50 (0.76, 2.94)	0.241
Oceania	1	-	-	1.05 (0.40, 2.76)	0.921
Europe	1	-	-	1.05 (0.32, 3.45)	0.936
Sample					
<500	4	0	0.508	1.65 (1.03, 2.63)	0.037
>500	1	-	-	0.96 (0.93, 0.99)	0.01
Study design					
Retrospective	2	76.2	0.04	1.29 (0.61, 2.73)	0.498
Prospective	3	0	0.436	1.41 (0.77, 2.57)	0.264
Diagnostic criteria of GDM					
IADPSG	1	-	-	1.05 (0.40, 2.76)	0.921
NDDG	1	-	-	2.40 (0.88, 6.58)	0.089
Others	2	0	0.333	1.73 (0.92, 3.25)	0.091
Diagnostic criteria of T2DM					
WHO	4	0	0,508	1.65 (1.03, 2.63)	0.037
NDDG	1	-	-	0.96 (0.93, 0.99)	0.01
***Progestin-only contraceptive***	3	61	0.077	2.12 (1.00, 4.45)	0.049
Location					
North America	2	75.6	0.043	1.84 (0.77, 4.40)	0.169
Europe	1	-	-	4.28 (0.90, 20.38)	0.068
Sample					
<500	1	-	-	4.28 (0.90, 20.38)	0.068
>500	2	75.6	0.043	1.84 (0.77, 4.40)	0.169
Study design					
Retrospective	1	-	-	2.87 (1.57, 5.26)	0.001
Prospective	2	55.9	0.132	1.83 (0.55, 6.03)	0.324
Diagnostic criteria of GDM					
NDDG	1	-	-	2.87 (1.57, 5.26)	0.001
Diagnostic criteria of T2DM					
NDDG	1	-	-	2.87 (1.57, 5.26)	0.001
ADA	2	55.9	0.132	1.83 (0.55, 6.03)	0.324
***Greater education***	4	62.7	0.045	0.53 (0.20, 1.37)	0.188
Location					
North America	1	-	-	0.46 (0.24, 0.88)	0.019
Asia	1	-	-	0.10 (0.02, 0.55)	0.008
Oceania	1	-	-	0.60 (0.24, 1.51)	0.278
Africa	1	-	-	4.60 (0.58, 36.61)	0.149
Sample					
<500	3	74.8	0.019	0.60 (0.10, 3.47)	0.566
>500	1	-	-	0.46 (0.24, 0.88)	0.019
Study design					
Retrospective	1	-	-	4.60 (0.58, 36.61)	0.149
Prospective	3	40.6	0.186	0.44 (0.26, 0.72)	0.015
Diagnostic criteria of GDM					
IADPSG	1	-	-	0.60 (0.24, 1.51)	0.278
Others	1	-	-	4.60 (0.58, 36.61)	0.149
Diagnostic criteria of T2DM					
**WHO**	**3**	74.8	0.019	0.60 (0.10, 3.47)	0.566

#### 3.3.2 Pregnancy-related factors

A meta-analysis was carried out on 13 pregnancy-related variables: early diagnosis of GDM (20, 23, 30, 33, 60), recurrence of GDM (20, 33, 37, 51), insulin use during pregnancy (17, 19, 20, 22, 26, 28–30, 34, 37, 40, 57, 58, 60), pre-pregnancy BMI (24, 25, 28–32, 47, 57, 59, 61), BMI during pregnancy (17, 19, 23, 35, 60), BMI after delivery (26, 34, 42, 47, 50, 58), weight change (18, 35, 60), gestational interval (35, 51), parity (26, 58), waist circumference (19, 24, 26, 61), macrosomia (26, 30, 35), neonatal birth weight (26, 33, 58), and hypertension (19, 34, 43, 61), which were reported in 5, 4, 14, 18, 3, 8, 3, 2, 2, 4, 3, 3, and 4 studies, respectively. Among the factors examined, six were identified as high-risk factors for the development of Type 2 diabetes (T2DM). These included: recurrence of gestational diabetes mellitus (GDM) (OR: 2.63, 95% CI: 1.88-3.69, P < 0.001), insulin use during pregnancy (OR: 4.35, 95% CI: 3.17-5.96, P < 0.001), pre-pregnancy BMI (OR: 2.97, 95% CI: 2.16-4.07, P < 0.001), BMI after delivery (OR: 4.17, 95% CI: 2.58-6.74, P < 0.001), macrosomia (OR: 3.30, 95% CI: 1.45-7.49, P = 0.004), and hypertension (OR: 5.19, 95% CI: 1.31-20.51, P = 0.019). Among the pregnancy-related variables, BMI during pregnancy was identified as a moderate-risk factor (OR: 1.06, 95% CI: 1.00-1.12, P = 0.056), though its significance was borderline. In contrast, several factors - weight change (OR: 1.03, 95% CI: 0.90-1.19, P = 0.647), gestational interval (OR: 1.68, 95% CI: 1.09-2.58, P = 0.018), parity (OR: 1.12, 95% CI: 0.84-1.49, P = 0.432), waist circumference (OR: 1.12, 95% CI: 0.98-1.29, P = 0.094), and neonatal birth weight (OR: 1.22, 95% CI: 0.74-2.00, P = 0.442) - were also classified as moderate-risk factors but did not reach statistical significance. An early diagnosis of GDM (OR: 0.96, 95% CI: 0.93-0.99, P = 0.26), as reported in five studies, was identified as a protective factor. For the meta-analysis, four factors - macrosomia, early diagnosis of GDM, recurrence of GDM, and gestational interval - were analyzed via a fixed-effects model due to low heterogeneity. The remaining nine factors were analyzed using a random-effects model due to significant heterogeneity. However, when comparing results from both models, only BMI during pregnancy and waist circumference showed significant differences, suggesting stability for the other factors. Even when studies related to BMI during pregnancy and waist circumference were excluded individually, the source of heterogeneity remained unclear. In this meta-analysis, weight change, parity, waist circumference, and neonatal birth weight were not significantly associated with the development of T2DM (P > 0.05). Notably, insulin use during pregnancy and hypertension were significantly correlated with publication bias (P < 0.05).

#### 3.3.3 Laboratory indicators

A meta-analysis was carried out on four laboratory parameters: HbA1c (20, 29, 30) (OR: 3.32, 95% CI=1.81-6.11, P<0.001), FBG (19, 20, 23, 26, 32–34, 37, 57, 58, 60) (OR: 1.58, 95% CI=1.36-1.84, P<0.001), OGTT 1-hour (32, 33, 58, 60) (OR: 1.38, 95% CI=1.02-1.87, P=0.037), and OGTT 2-hour (19, 20, 29, 30, 32, 57, 58, 60) (OR: 1.54, 95% CI=1.28-1.58, P<0.001). These parameters were analyzed across three, twelve, four, and eight studies, respectively. HbA1c was identified as a high-risk factor for T2DM, while the other three parameters were classified as moderate-risk factors. Given the low heterogeneity observed for HbA1c, a fixed-effects model was applied. In contrast, the other three factors demonstrated high heterogeneity (I² = 96%, 99.5%, 80%), prompting the use of a random-effects model. Our findings remained consistent and robust after adjusting for the fixed-effects model, with all four factors showing a significant association with the occurrence of T2DM (P < 0.05). Furthermore, a significant publication bias was identified for the 2-hour OGTT (P < 0.01).

#### 3.3.4 Subgroup analysis and sensitivity analyses

Subgroup analyses suggested that regional differences, diagnostic criteria, sample size, and study design may contribute to the observed heterogeneity in factors such as age, insulin use during pregnancy, and FBG. Specifically, sample size appeared to be a key source of heterogeneity for hypertension and the 1-hour OGTT, while variations in study design could explain the heterogeneity observed in waist circumference and the early diagnosis of GDM. Despite these sources of heterogeneity, sensitivity analyses confirmed the robustness and reliability of our findings. The results of the sensitivity analysis are presented in **Supplementary Figure S1**.

## 4 Discussion

46 studies encompassing 196,494 patients were ultimately included in our research. Multiple risk factors in the progression from GDM to T2DM were systematically evaluated. Our findings reveal that several factors significantly contribute to this progression, including age, family history of diabetes, use of progestin-only contraceptives, recurrence of GDM, insulin use during pregnancy, pre- and post-pregnancy BMI, macrosomia, hypertension, and persistently elevated levels of HbA1c, FBG, one-hour and two-hour OGTT results. These results highlight the importance of continuous monitoring and early intervention for high-risk GDM patients in clinical practice.

More evidence suggests that the progression of GDM to T2DM may be significantly correlated with insulin β-cell dysfunction (62). In the forthcoming discussion, this study will delve into the mechanism underlying the role of three distinct types of risk factors in the transition from GDM to T2DM.

### 4.1 Demographic and lifestyle characteristics

Our study revealed that women with GDM who used progestin-only contraceptives were of advanced maternal age, or had a family history of diabetes were more likely to develop T2DM. These findings are consistent with those of Rayanagoudar et al., who also identified family history and advanced maternal age as significant risk factors for T2DM (63). They reported an RR of 1.70 for a positive family history of T2DM, which is consistent with the OR of 1.47 observed in our study. This indicates that familial factors contribute to a higher incidence of T2DM among women with a history of GDM, to some extent. The potential underlying reasons may include shared lifestyles and life philosophies within families (63). Moreover, our findings indicated a lower risk of T2DM in Caucasian women, aligning with those of Rayanagoudar et al. and You et al., who reported higher risks in Black and non-Hispanic White women after GDM (10, 63). It is important to note that, while race was not identified as a significant risk factor in our study, the observed heterogeneity in the analysis may still reflect variations in lifestyle and genetic factors across different racial groups.

Overall, patient age is a significant risk factor. As individuals age, the function of pancreatic β-cells typically declines, which directly impacts the synthesis and secretion of insulin, thereby influencing glucose regulation (64, 65). For older patients with GDM, β-cell function may have already been impaired due to aging (66, 67). The hyperglycemic stress experienced during pregnancy can further accelerate this decline, which increases their risk of developing T2DM after childbirth. Aging is also associated with heightened insulin resistance (68, 69). As individuals age, they typically experience a reduction in muscle mass and changes in visceral fat distribution, both of which contribute to systemic insulin resistance (70–72). Moreover, advancing age often coexists with inflammaging, which is a state of chronic low-grade inflammation (73, 74). Elevated levels of inflammatory markers, such as tumor necrosis factor-α (TNF-α) and interleukin-6 (IL-6), exacerbate insulin resistance and further impair β-cell function (75, 76). Lastly, although there remains ongoing debate about the link between progestin-only contraceptives and the development of diabetes, this study has found that women who use these contraceptives appear to have an increased risk of developing T2DM. Progestin-only contraceptives may contribute to this risk by inducing apoptosis in pancreatic β-cells (77), which affects blood glucose levels and disrupts glucose metabolism. This disruption can, in turn, lead to increased insulin resistance and facilitate the progression from GDM to T2DM (78, 79).

### 4.2 Pregnancy-related factors

Our findings suggest that several pregnancy-related factors significantly elevate the risk of developing T2DM in women with GDM. These factors include GDM recurrence, insulin use during pregnancy, higher BMI before or after pregnancy, the delivery of macrosomic infants, and the presence of hypertension. These results are consistent with those of Rayanagoudar et al., particularly regarding BMI and insulin use during pregnancy (63). Both studies demonstrate that a high BMI substantially increases the risk of T2DM. Rayanagoudar et al. reported a progressive increase in T2DM risk with rising BMI, particularly when BMI reaches overweight or obese levels (63). This underscores the importance of managing weight before, during, and after pregnancy to prevent the progression from GDM to T2DM. Moreover, insulin use during pregnancy and GDM recurrence were identified as significant independent risk factors for T2DM. Rayanagoudar et al. found that women with GDM who required insulin therapy had a notably higher risk of developing T2DM (63). This may reflect the degree of β-cell dysfunction and the dependency on insulin during and after pregnancy. Lastly, in line with the findings of Rayanagoudar et al., our study also indicates no significant association between breastfeeding, neonatal birth weight, and the risk of T2DM in female GDM patients.

Increased insulin requirements in GDM are indicative of β-cell dysfunction. However, when β-cells cannot meet the heightened demand, GDM may develop (80). Notably, GDM patients who require exogenous insulin therapy may already have significant β-cell impairment (80). The compromised β-cell function may not fully recover postpartum, thereby raising the risk of progressing to T2DM. Additionally, a high BMI plays a crucial role in the development of insulin resistance. Obesity leads to an overproduction of inflammatory cytokines, such as TNF-α and IL-6, in adipose tissue, further exacerbating insulin resistance (81–83). Therefore, a high BMI is a significant risk factor in the progression from T2DM to GDM. Pregnancy-related complications, such as hypertension, are also important high-risk factors. Hypertension is often a marker of underlying endothelial dysfunction and systemic inflammation, both of which are closely linked to insulin resistance and the development of diabetes (84, 85). Lastly, macrosomia is another significant risk factor for the transition from GDM to T2DM, involving complex biological and physiological mechanisms. Macrosomia is typically the result of poor glycemic control during pregnancy. In GDM, insulin resistance and/or insufficient β-cell secretion lead to elevated maternal glucose levels (86, 87). These elevated glucose levels can cross the placenta, stimulate fetal growth, and lead to excessive fetal weight, or macrosomia (87). Macrosomia not only reflects the increased fetal size but also mirrors the mother’s metabolic state and insulin sensitivity (86, 87). Furthermore, the development of GDM and macrosomia is correlated with inflammation and oxidative stress (88–90). A hyperglycemic environment can promote the production of free radicals and the release of inflammatory cytokines, which in turn may damage β-cells and impair their function (88–90).

### 4.3 Laboratory indicators

In terms of laboratory indicators, this study examined the correlation of HbA1c, FBG, as well as the one-hour and two-hour values of OGTT with the progression from GDM to T2DM. All of these indicators were proven to be significantly linked to an increased risk of developing T2DM. These parameters are crucial for monitoring glycemic control in diabetic patients, and the study’s findings further highlight their importance in predicting the progression from GDM to T2DM.

HbA1c, as a critical marker of long-term glycemic control, is particularly crucial in managing GDM. Its low heterogeneity across studies suggests that HbA1c consistently predicts T2DM risk, and can serve as a stable and reliable potential risk assessment tool. For GDM patients, persistently elevated HbA1c levels reflect prolonged hyperglycemia and may indicate further deterioration of pancreatic β-cell function (91, 92). This sustained hyperglycemic state can enhance insulin resistance, impose a greater burden on β-cells, and ultimately lead to β-cell exhaustion (91, 92). Furthermore, the analysis of FBG suggests a moderate increase in the risk of developing T2DM (OR = 1.58) and shows the importance of continued monitoring of FBG levels after pregnancy. As a tool for routine monitoring, FBG immediately reflects glycemic control and aids in the early identification of GDM patients who may develop T2DM. FBG is primarily regulated by the balance between hepatic glucose production and insulin release from pancreatic β-cells (93). For GDM patients, if β-cells fail to manage the persistent hyperglycemic stress after childbirth, their function may continue declining and lead to sustained elevations in FBG levels, which may result in the development of T2DM from GDM (94). Finally, the ORs for OGTT at one hour and two hours were 1.38 and 1.54, respectively, indicating the potential of OGTT in predicting T2DM risk. OGTT can be employed to assess insulin secretion and sensitivity by measuring an individual’s glycemic response to oral glucose (95, 96). Elevated OGTT values at one and two hours generally indicate insufficient insulin secretion or impaired insulin action (95, 96). These elevated test results physiologically reflect a diminished β-cell response to glucose and inadequate peripheral tissue response to insulin, serving as an early warning signal for T2DM development (95, 96). Therefore, systematic postpartum glycemic monitoring is essential for GDM patients, particularly those with high HbA1c and FBG levels. Regular OGTTs complement routine FBG monitoring by identifying glycemic abnormalities that may otherwise go unnoticed, facilitating early detection and timely intervention for T2DM risk.

### 4.4 Subgroup analysis and sensitivity analyses

Lastly, subgroup and sensitivity analyses were performed to delve into the prevalence and risk factors for T2DM in female GDM patients. The subgroup analysis of prevalence revealed that the incidence of T2DM among GDM patients in Asia is slightly lower compared to Europe, the Americas, and Oceania. This discrepancy may be attributed to differences in sample sizes and diagnostic criteria across regions. Additionally, the subgroup analysis of risk factors highlighted that regional variations and differences in diagnostic standards could explain the observed heterogeneity in age, insulin use during pregnancy, and FBG levels. These findings are consistent with the results of Rayanagoudar et al., who identified that follow-up duration significantly influences the risk assessment of FBG, BMI, and insulin use (63). This suggests that researchers should consider region-specific medical practices and diagnostic criteria when studying T2DM risk factors in different global regions. The sample size was identified as a source of heterogeneity for hypertension and the 1-hour OGTT, while the study design contributed to the heterogeneity observed in waist circumference and early GDM diagnosis. This highlights the critical role of study design in interpreting research findings, as variations in sampling and data collection methods can lead to biased conclusions.

Despite the aforementioned heterogeneity, sensitivity analysis confirms the stability of our findings, which are consistent with those of Rayanagoudar et al. They reported that the impact of certain key variables, such as FBG and BMI, remained significant despite variations in follow-up duration (63). This shows the reliability of the identified risk factors for the progression from GDM to T2DM, even in the presence of potential biases. Additionally, the study highlighted the potential influence of various factors on T2DM development in pregnant women, including healthful dietary patterns, physical activity, sedentary behaviors, habitual iron intake, alcohol consumption, coffee consumption, and multiple pregnancies. However, due to the limited number of original studies, the existing data are insufficient to conduct a meta-analysis on the precise effects of these factors on T2DM risk. Therefore, further research is needed to delve into these associations. It is worth noting that, in terms of the prevention of T2DM, integrative medicine research has indicated that natural remedies such as ginger and Ganoderma lucidum (lingzhi), along with their extracts, may be therapeutic and safe in modulating human metabolism (97–99). These natural agents merit further exploration as promising research foci in future studies.

## 5 Limitations

Although our meta-analysis aimed to integrate and analyze data from multiple studies, significant differences existed in the diagnostic criteria for GDM and T2DM across the selected studies. This heterogeneity may have affected the consistency and generalizability of the results, thereby limiting our ability to statistically assess these risk factors. Additionally, some studies may not report all essential statistical data, such as CIs, standard deviations, or specific p-values, which could have introduced imprecision in the analyses. Despite our efforts to include as many studies as possible, the sample size in some subgroup analyses remained relatively small, which potentially increases the influence of chance factors. Moreover, the geographic distribution and demographic characteristics of the included studies may not fully reflect the broader population, further limiting the generalizability of our findings.

## 6 Conclusion

This study has identified several significant risk factors correlated with the development of T2DM in female GDM patients. These factors include the use of progestin-only contraceptives, recurrence of GDM, insulin use during pregnancy, pre- and post-pregnancy BMI, macrosomia, hypertension, and persistently elevated levels of HbA1c, FBG, as well as 1-hour and 2-hour OGTT readings. These findings offer robust and reliable evidence that can guide the management of T2DM in this population. The results have significant implications for health management, as well as for clinical T2DM prevention and intervention in pregnant women. Clinicians can tailor interventions to address these risk factors, ultimately reducing the incidence of T2DM and improving the clinical outcomes in women with GDM.

## Data Availability

The original contributions presented in the study are included in the article/**Supplementary Material**. Further inquiries can be directed to the corresponding authors.

## Supplementary material

Supplementary Figure 1 Sensitivity analysis of forest plots of comparison of the two models.

Supplementary Table 1 Literature search strategy.

Supplementary Table 2 Publication bias.

Supplementary Table 3 Study quality assessment using the Newcastle-Ottawa scale tool and AHRQ assessment tool.
