# Supplementary material for: Prevalence and risk factors for type 2 diabetes mellitus in women with gestational diabetes mellitus: a systematic review and meta-analysis
Source: Front Endocrinol (Lausanne). 2024 Dec 23;15:1486861. doi: 10.3389/fendo.2024.1486861 (PMC11700824; doi:10.3389/fendo.2024.1486861)
Supplement: Supplementary Table 1 — Literature search strategy. [file Table1.docx]

**Table S1.**Literature search strategy

| **Pubmed** | ("diabetes mellitus, type 2"[MeSH Terms] OR ("ketosis resistant diabetes mellitus"[Title/Abstract] OR "non insulin dependent diabetes mellitus"[Title/Abstract] OR "stable diabetes mellitus"[Title/Abstract] OR "NIDDM"[Title/Abstract] OR "MODY"[Title/Abstract] OR ("Slow-Onset"[All Fields] AND "diabetes mellitus"[Title/Abstract]) OR "noninsulin dependent diabetes mellitus"[Title/Abstract] OR "maturity onset diabetes"[Title/Abstract] OR "maturity onset diabetes"[Title/Abstract] OR "type 2 diabetes"[Title/Abstract] OR "adult onset diabetes mellitus"[Title/Abstract] OR "non insulin dependent diabetes mellitus"[Title/Abstract] OR "adult onset diabetes"[Title/Abstract] OR "diabetes mellitus type 2"[Title/Abstract] OR "diabetes mellitus type ii"[Title/Abstract] OR "diabetes type 2"[Title/Abstract] OR "diabetes type ii"[Title/Abstract] OR "dm 2"[Title/Abstract] OR "insulin independent diabetes"[Title/Abstract] OR "ketosis resistant diabetes mellitus"[Title/Abstract] OR "non insulin dependent diabetes"[Title/Abstract] OR "noninsulin dependent diabetes"[Title/Abstract] OR "T2DM"[Title/Abstract] OR "type ii diabetes"[Title/Abstract])) AND ("diabetes, gestational"[MeSH Terms] OR ("diabetes gestational"[Title/Abstract] OR "diabetes pregnancy induced"[Title/Abstract] OR "pregnancy induced diabetes"[Title/Abstract] OR "gestational diabetes"[Title/Abstract] OR (("diabetes mellitus"[MeSH Terms] OR ("Diabetes"[All Fields] AND "Mellitus"[All Fields]) OR "diabetes mellitus"[All Fields]) AND "gravidarum"[Title/Abstract]) OR (("diabetes mellitus"[MeSH Terms] OR ("Diabetes"[All Fields] AND "Mellitus"[All Fields]) OR "diabetes mellitus"[All Fields]) AND "of pregnancy"[Title/Abstract]) OR (("diabete"[All Fields] OR "diabetes mellitus"[MeSH Terms] OR ("Diabetes"[All Fields] AND "Mellitus"[All Fields]) OR "diabetes mellitus"[All Fields] OR "Diabetes"[All Fields] OR "diabetes insipidus"[MeSH Terms] OR ("Diabetes"[All Fields] AND "insipidus"[All Fields]) OR "diabetes insipidus"[All Fields] OR "diabetic"[All Fields] OR "diabetics"[All Fields] OR "diabets"[All Fields]) AND "of pregnancy"[Title/Abstract]) OR "maternal gestational diabetes mellitus"[Title/Abstract] OR "pregnancy diabetes"[Title/Abstract])) 4698 |
| --- | --- |
| **Web of Science** | 1: Diabetes Mellitus, Type 2 OR Ketosis-Resistant Diabetes Mellitus OR Non-Insulin-Dependent Diabetes Mellitus OR Stable Diabetes Mellitus OR NIDDM OR MODY OR Slow-Onset Diabetes Mellitus OR Noninsulin-Dependent Diabetes Mellitus OR Maturity-Onset Diabetes OR Maturity Onset Diabetes OR Type 2 Diabetes OR Adult-Onset Diabetes Mellitus OR non insulin dependent diabetes mellitus OR adult onset diabetes OR diabetes mellitus type 2 OR diabetes mellitus type ii OR diabetes type 2 OR diabetes type II OR dm 2 OR insulin independent diabetes OR ketosis resistant diabetes mellitus OR non insulin dependent diabetes OR noninsulin dependent diabetes OR T2DM OR type II diabetes Tue Mar 12 2024 01:20:06 GMT+0800 Result 225225  2: ((((((((TS=(Diabetes, Gestational)) OR TS=(Diabetes, Pregnancy Induced)) OR TS=(Pregnancy-Induced Diabetes)) OR TS=(gestational diabetes)) OR TS=(diabetes mellitus gravidarum)) OR TS=(diabetes mellitus of pregnancy)) OR TS=(diabetes of pregnancy)) OR TS=(maternal gestational diabetes mellitus)) OR TS=(pregnancy diabetes) Tue Mar 12 2024 01:27:58 GMT+0800 Result: 27861  3: #2 AND #1 Tue Mar 12 2024 01:30:08 GMT+0800 Result: 6327 |
| **Cochrane** | ID Search  #1 MeSH descriptor: [Diabetes Mellitus, Type 2] explode all trees  #2 (Diabetes Mellitus, Type 2):ti,ab,kw OR (Diabetes Mellitus, Noninsulin-Dependent):ti,ab,kw OR (Ketosis-Resistant Diabetes Mellitus):ti,ab,kw OR (Non-Insulin-Dependent Diabetes Mellitus):ti,ab,kw OR (Stable Diabetes Mellitus):ti,ab,kw OR (Diabetes Mellitus, Type II):ti,ab,kw OR (NIDDM):ti,ab,kw OR (Diabetes Mellitus, Noninsulin Dependent):ti,ab,kw OR (Diabetes Mellitus, Maturity-Onset):ti,ab,kw OR (Diabetes Mellitus, Maturity Onset):ti,ab,kw OR (MODY):ti,ab,kw OR (Diabetes Mellitus, Slow-Onset):ti,ab,kw OR (Diabetes Mellitus, Slow Onset):ti,ab,kw OR (Noninsulin-Dependent Diabetes Mellitus):ti,ab,kw OR (Noninsulin Dependent Diabetes Mellitus):ti,ab,kw OR (Maturity-Onset Diabetes):ti,ab,kw OR (Maturity Onset Diabetes):ti,ab,kw OR (Type 2 Diabetes):ti,ab,kw OR (Adult-Onset Diabetes Mellitus):ti,ab,kw OR (Diabetes Mellitus, Adult Onset):ti,ab,kw OR (non insulin dependent diabetes mellitus):ti,ab,kw OR (adult onset diabetes):ti,ab,kw OR (diabetes mellitus type 2):ti,ab,kw OR (diabetes mellitus type ii):ti,ab,kw OR (diabetes mellitus, non-insulin-dependent):ti,ab,kw OR (diabetes type 2):ti,ab,kw OR (diabetes type II):ti,ab,kw OR (dm 2):ti,ab,kw OR (insulin independent diabetes):ti,ab,kw OR (ketosis resistant diabetes mellitus):ti,ab,kw OR (maturity onset diabetes):ti,ab,kw OR (NIDDM):ti,ab,kw OR (non insulin dependent diabetes):ti,ab,kw OR (non-insulin-dependent diabetes mellitus):ti,ab,kw OR (noninsulin dependent diabetes):ti,ab,kw  OR (T2DM):ti,ab,kw  #3 #1 OR #2  #4 MeSH descriptor: [Diabetes, Gestational] explode all trees  #5 (Diabetes, Gestational):ti,ab,kw OR (Diabetes, Pregnancy-Induced):ti,ab,kw OR (Diabetes, Pregnancy Induced):ti,ab,kw OR (gestational diabetes):ti,ab,kw OR (diabetes mellitus gravidarum):ti,ab,kw OR (diabetes of pregnancy):ti,ab,kw OR (diabetes, gestational):ti,ab,kw OR (diabetes, pregnancy):ti,ab,kw OR (maternal gestational diabetes mellitus):ti,ab,kw OR (pregnancy diabetes):ti,ab,kw  #6 #4 OR #5  #7 #3 AND #6  2212 |
| **Embase** | #7. #3 AND #6 9281  #6. #4 OR #5 56504  #5. 'gestational diabetes':ab,ti OR 'diabetes mellitus gravidarum':ab,ti OR 'diabetes mellitus of pregnancy':ab,ti OR 'diabetes of pregnancy':ab,ti OR 'maternal gestational diabetes mellitus':ab,ti OR 'pregnancy diabetes':ab,ti OR 'diabetes, gestational':ab,ti OR 'diabetes, pregnancy induced':ab,ti OR 'pregnancy-induced diabetes':ab,ti 35164  #4. 'gestational diabetes'/exp 52970  #3. #1 OR #2 412675  #2. 'non insulin dependent diabetes mellitus':ab,ti OR 'adult onset diabetes':ab,ti OR 'diabetes mellitus type 2':ab,ti OR 'diabetes mellitus type ii':ab,ti OR 'diabetes type 2':ab,ti OR 'diabetes type ii':ab,ti OR 'dm 2':ab,ti OR 'insulin independent diabetes':ab,ti OR 'ketosis resistant diabetes mellitus':ab,ti OR 'non insulin dependent diabetes':ab,ti OR 'noninsulin dependent diabetes':ab,ti OR t2dm:ab,ti OR 'type ii diabetes':ab,ti OR 'adult-onset diabetes mellitus':ab,ti OR 'type 2 diabetes':ab,ti OR 'maturity onset diabetes':ab,ti OR 'maturity-onset diabetes':ab,ti OR 'noninsulin-dependent diabetes mellitus':ab,ti OR 'slow-onset diabetes mellitus':ab,ti OR mody:ab,ti OR niddm:ab,ti OR 'stable diabetes mellitus':ab,ti OR 'non-insulin-dependent diabetes mellitus':ab,ti OR 'ketosis-resistant diabetes mellitus':ab,ti OR 'diabetes mellitus, type 2':ab,ti 308984  #1. 'non insulin dependent diabetes mellitus'/exp 353819 |
