# Supplementary material for: Prevalence and risk factors for type 2 diabetes mellitus in women with gestational diabetes mellitus: a systematic review and meta-analysis
Source: Front Endocrinol (Lausanne). 2024 Dec 23;15:1486861. doi: 10.3389/fendo.2024.1486861 (PMC11700824; doi:10.3389/fendo.2024.1486861)
Supplement: Supplementary Table 2 — Publication bias. [file Table2.docx]

**Table S2.**Funnel plots of Publication bias

Figure 1 African American


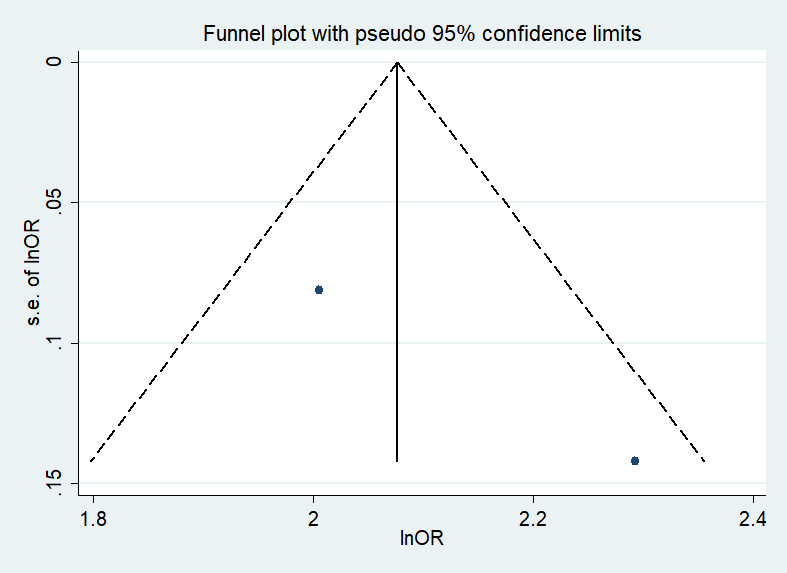


Figure 2 Age


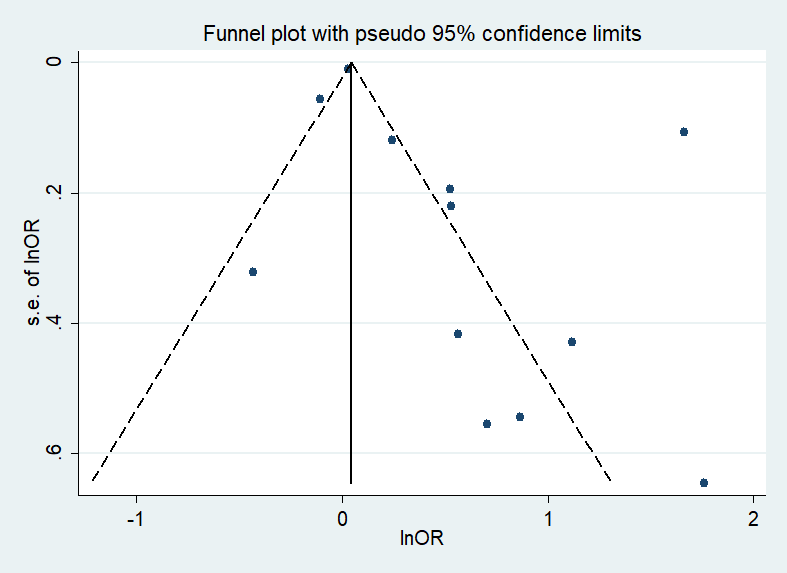


Figure 3 Asian


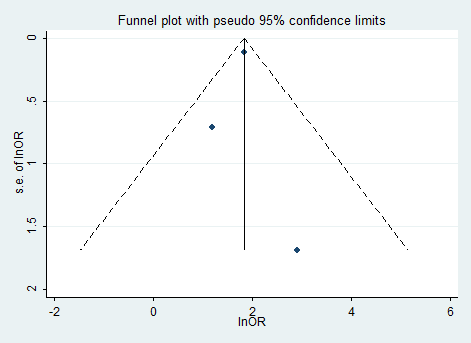


Figure 4 BMI after delivery


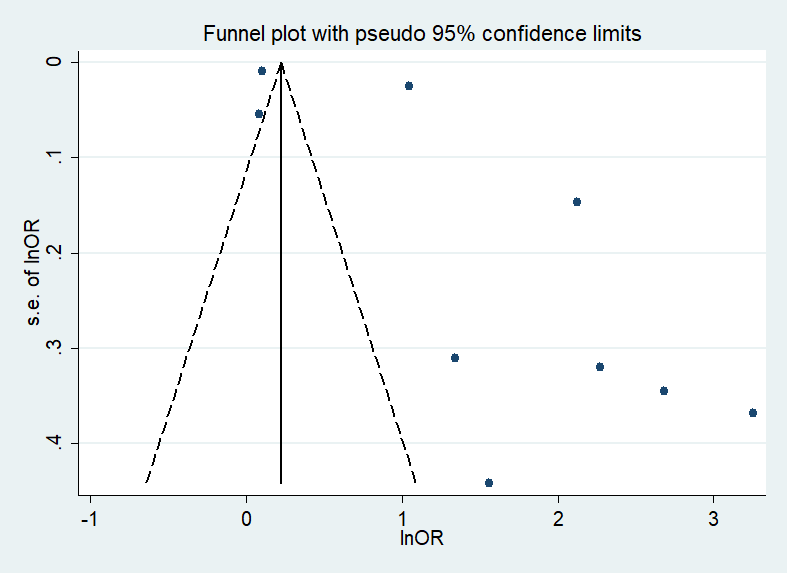


Figure 5 BMI in pregnancy


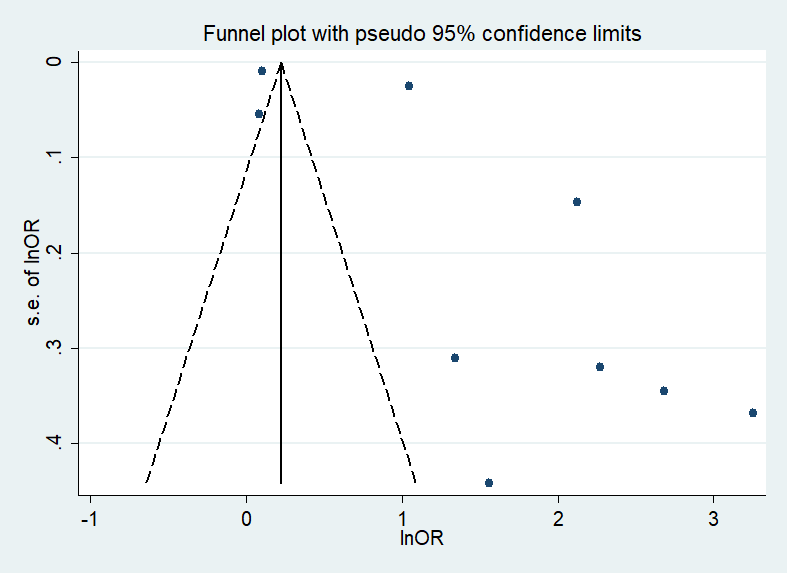


Figure 6 Breastfeeding


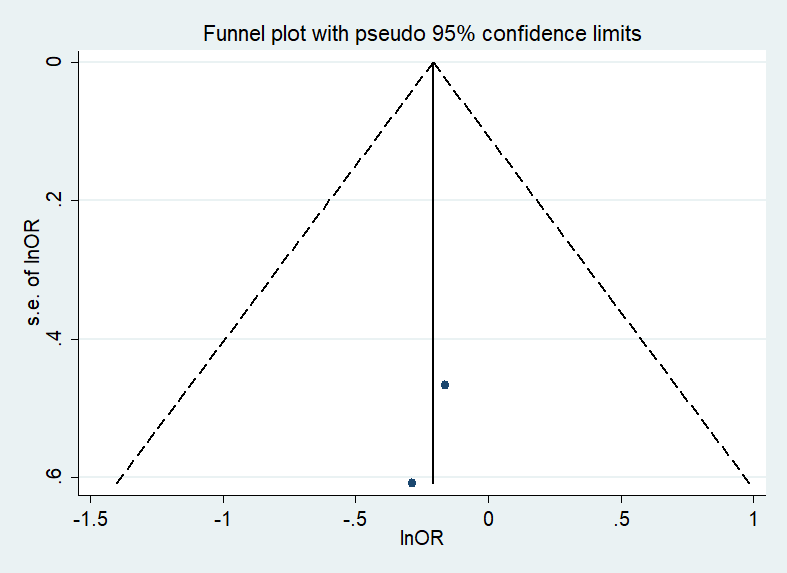


Figure 7 Early diagnosis GDM


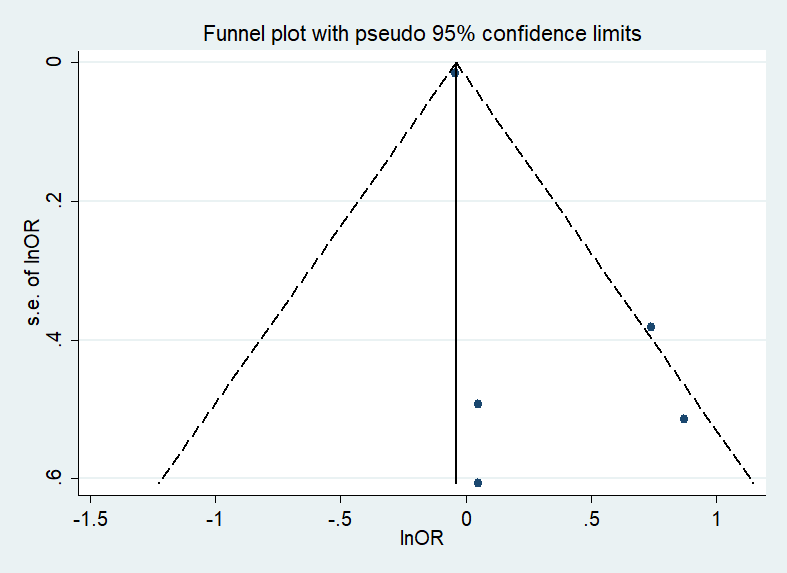


Figure 8 Family history of diabetes


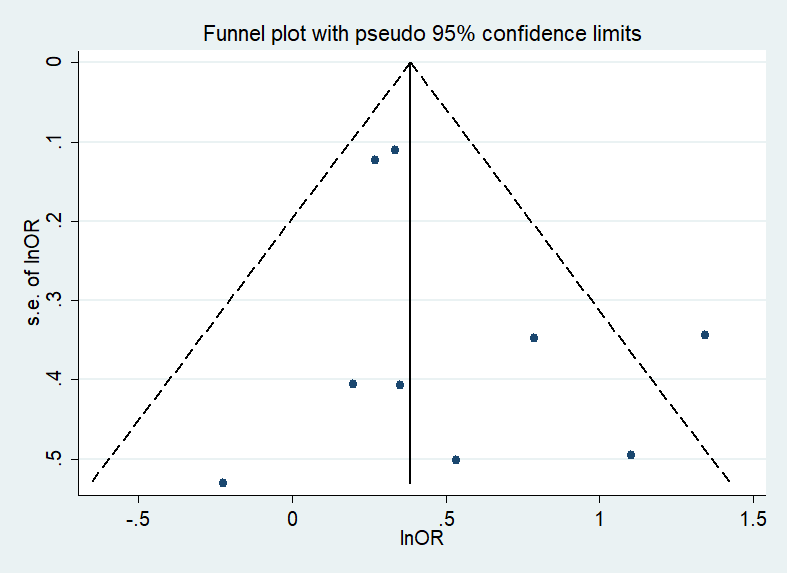


Figure 9 FBG


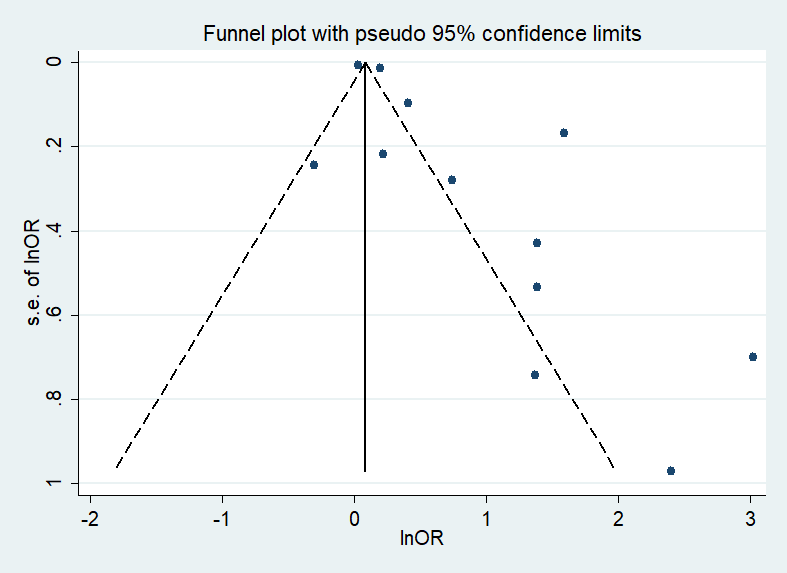


Figure 10 GDM recurrence


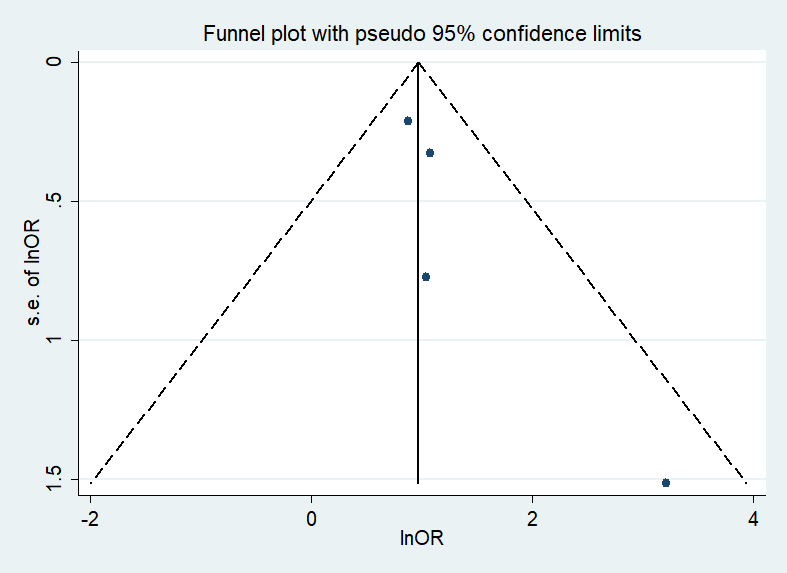


Figure 11 Gestational interval


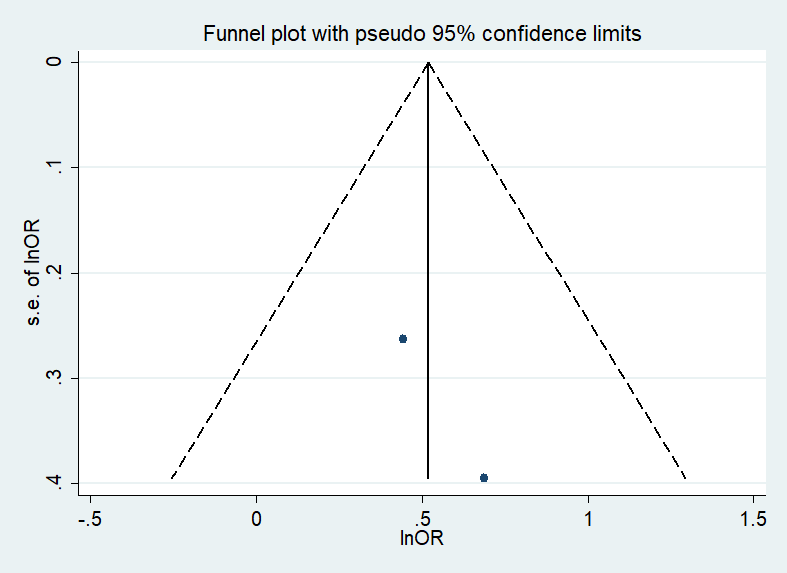


Figure 12 Greater education


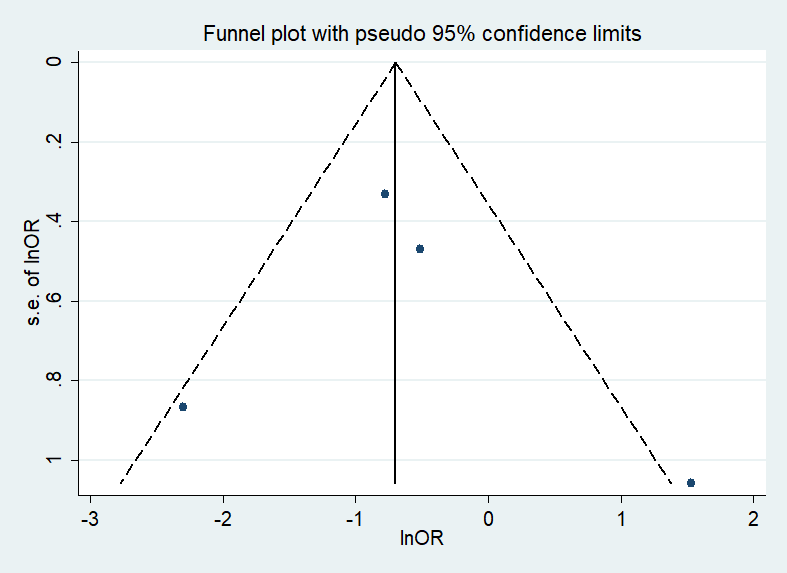


Figure 13 HbA1c


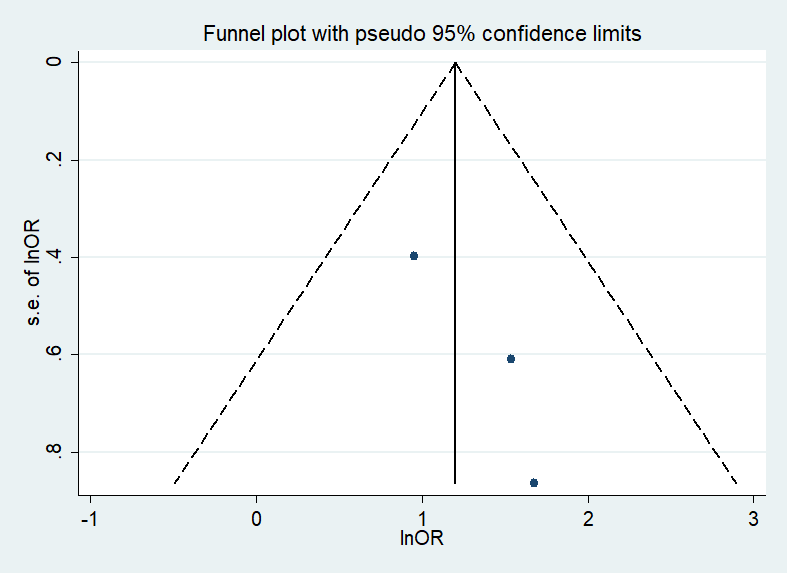


Figure 14 Hispanic


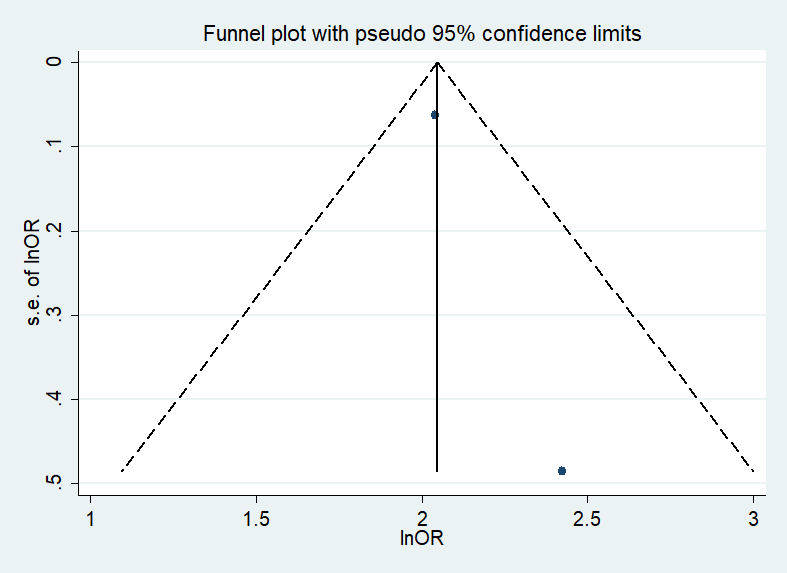


Figure 15 Hypertension


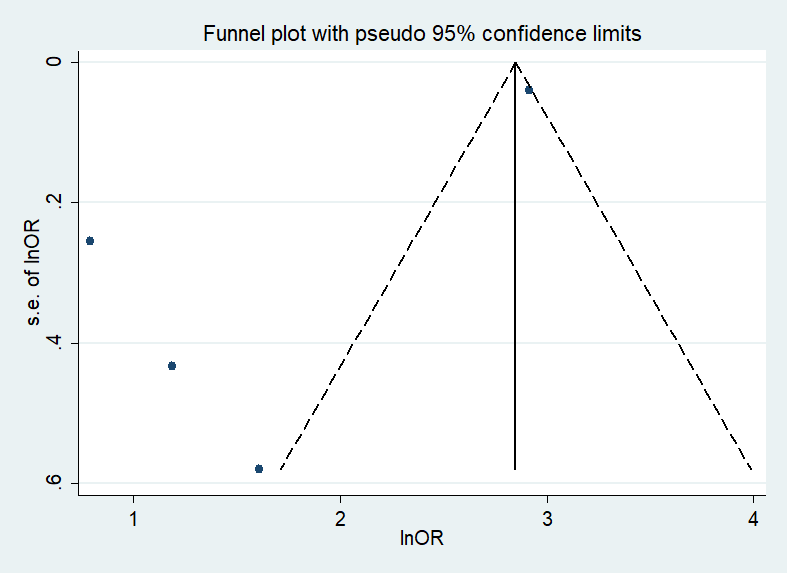


Figure 16 Insulin use in pregnancy


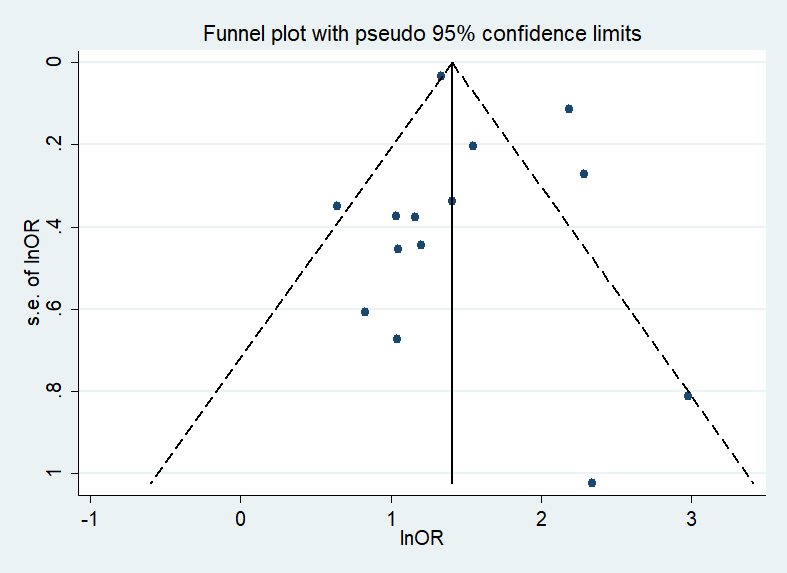


Figure 17 Macrosomia


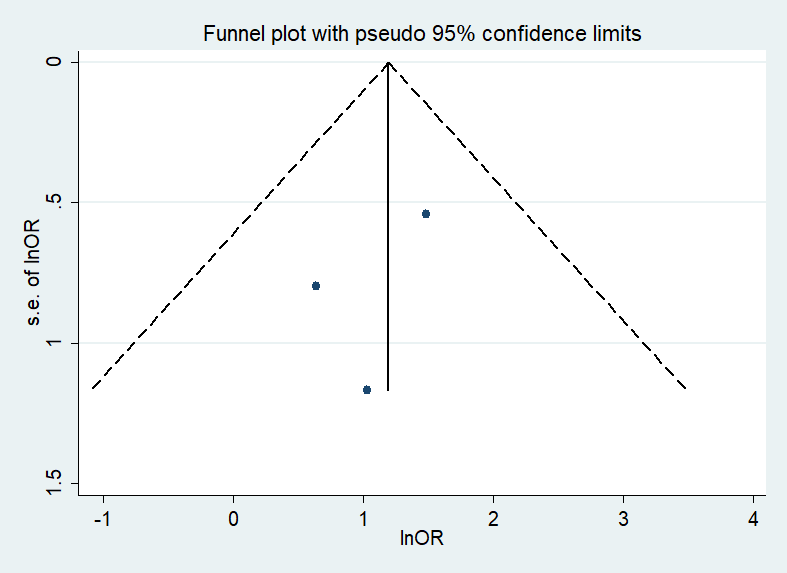


Figure 18 Neonatal birth weight


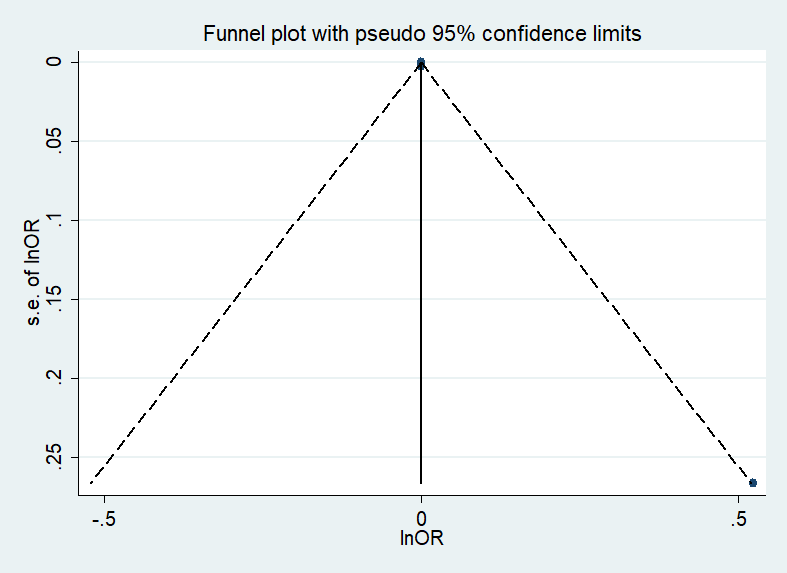


Figure 19 OGTT-1h


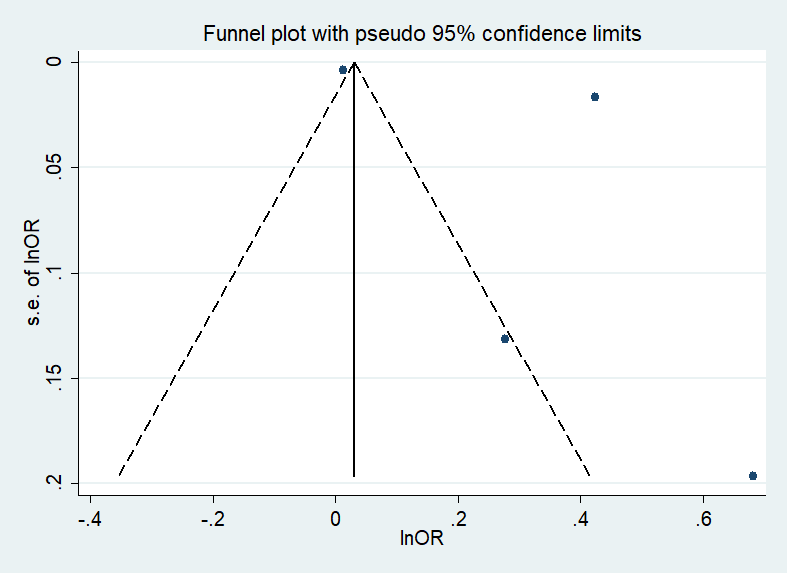


Figure 20 OGTT-2h


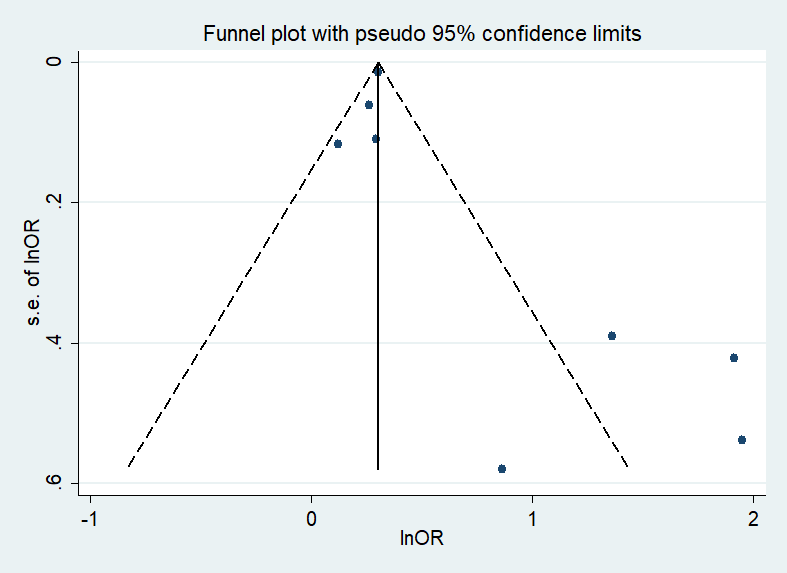


Figure 21 Parity


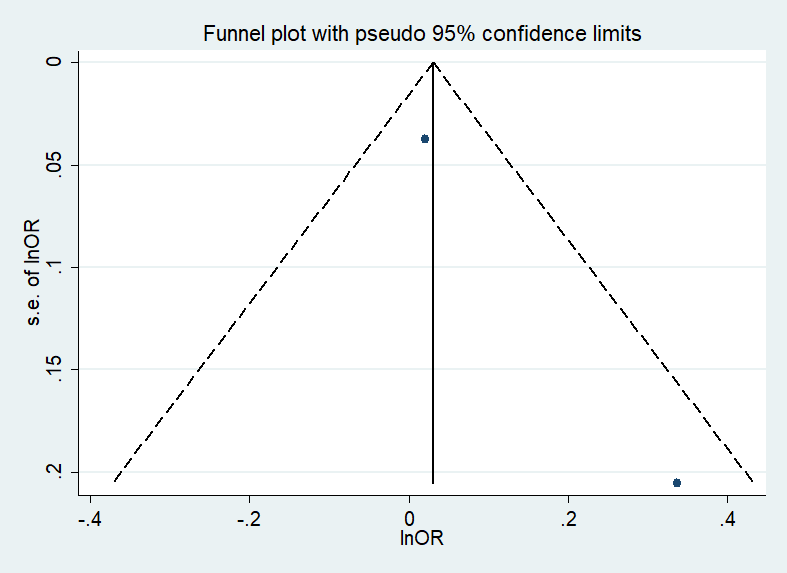


Figure 22 Pre-pregnancy BMI


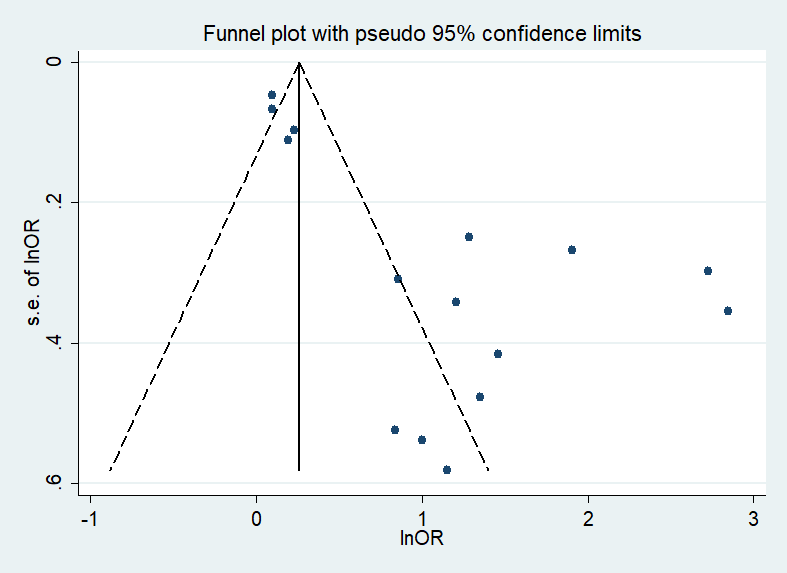


Figure 23 Use of progestin-only contraceptive


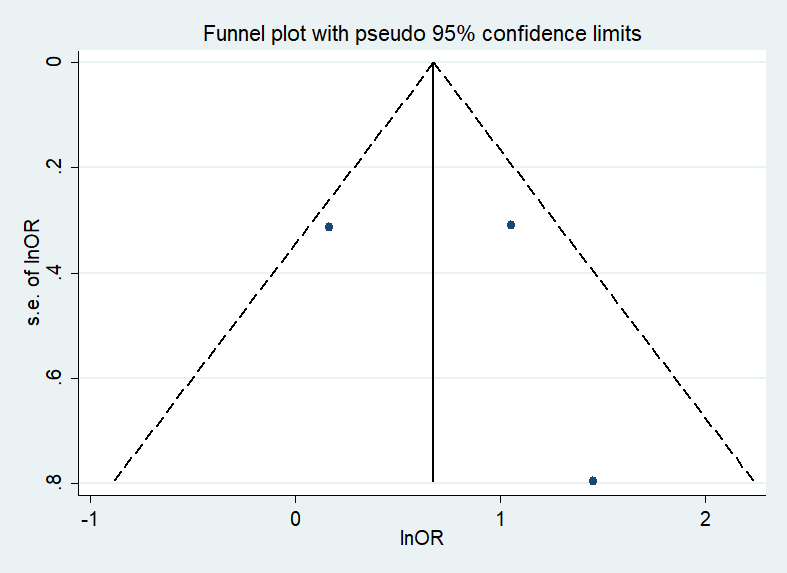


Figure 24 Waist circumference


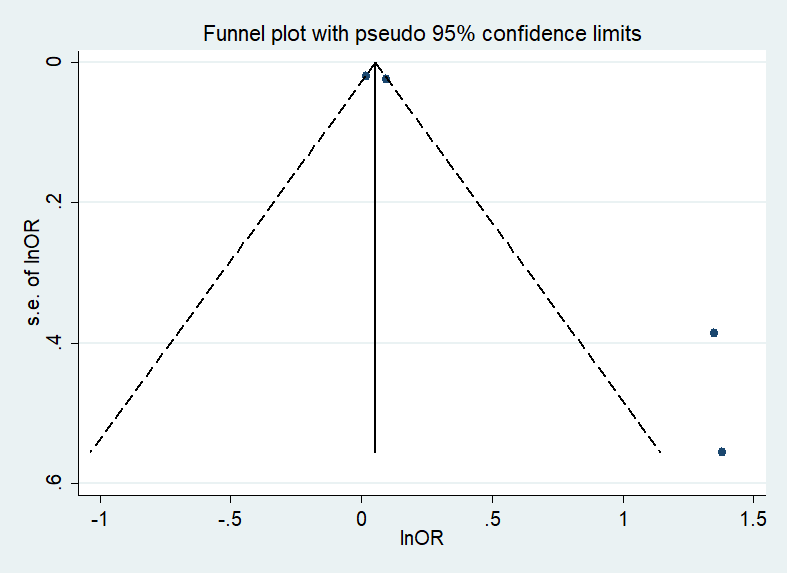


Figure 25 Weight change


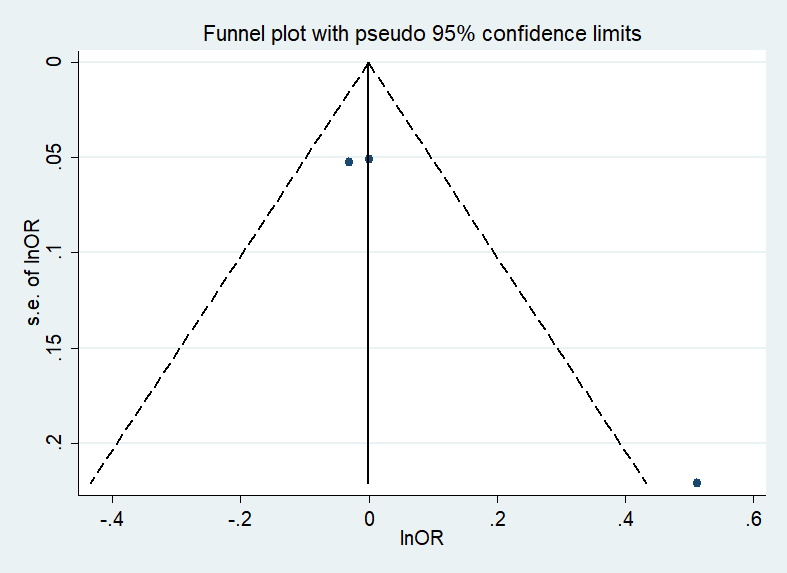


Figure 26 White


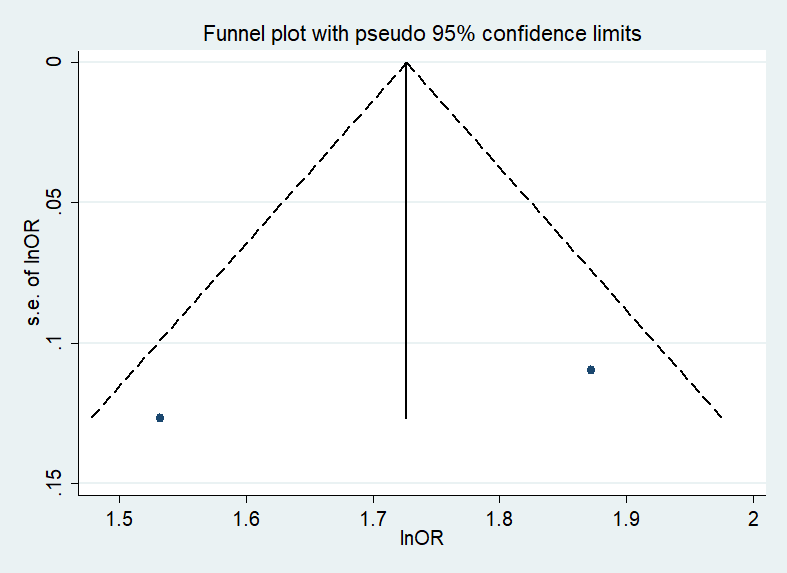


**Publication bias**

| **Risk factors** | **Begg's test** | | **Egger's test** | |
| --- | --- | --- | --- | --- |
|  | **z** | ***p*** | ***t*** | ***p*** |
| Weight change | 0.00 | 1.000 | 4.83 | 0.130 |
| Parity | 0.00 | 1.000 | - | - |
| Age | 0.89 | 0.373 | 0.11 | 0.916 |
| Waist circumference | 1.02 | 0.308 | -0.55 | 0.640 |
| Macrosomia | 0.00 | 1.000 | -0.87 | 0.546 |
| Neonatal birth weight | 0.00 | 1.000 | - | - |
| Family history of diabetes | -0.10 | 1.000 | 1.36 | 0.217 |
| Insulin use in pregnancy | 0.11 | 0.913 | 15.09 | 0.000 |
| Early diagnosis GDM | -0.24 | 1.000 | -3.06 | 0.055 |
| GDM recurrence | 1.02 | 0.308 | 3.23 | 0.084 |
| Hypertension | -0.34 | 1.000 | 18.47 | 0.003 |
| Progestin-only contraceptive | 0.00 | 1.000 | 0.49 | 0.712 |
| Breastfeeding | 0.00 | 1.000 | - | - |
| Greater education | 0.34 | 0.734 | 0.26 | 0.822 |
| Gestational interval | 0.00 | 1.000 | - | - |
| HbA1c | 0.00 | 1.000 | - | - |
| FBG | 0.07 | 0.945 | 1.50 | 0.164 |
| OGTT 1-h | 0.34 | 0.734 | 1.10 | 0.386 |
| OGTT 2-h | 1.11 | 0.266 | 9.30 | 0.000 |
| Pre-pregnancy BMI | 0.99 | 0.322 | -1.23 | 0.240 |
| BMI in pregnancy | 0.24 | 0.806 | -0.05 | 0.965 |
| BMI after delivery | 0.31 | 0.754 | 0.82 | 0.440 |
| Asian | 0.00 | 1.000 | 10.37 | 0.061 |
| White | 0.00 | 1.000 | - | - |
| Hispanic | 0.00 | 1.000 | - | - |
| African American | 0.00 | 1.000 | - | - |
