# Supplementary material for: Prevalence and risk factors for type 2 diabetes mellitus in women with gestational diabetes mellitus: a systematic review and meta-analysis
Source: Front Endocrinol (Lausanne). 2024 Dec 23;15:1486861. doi: 10.3389/fendo.2024.1486861 (PMC11700824; doi:10.3389/fendo.2024.1486861)
Supplement: Supplementary Table 3 — Study quality assessment using the Newcastle-Ottawa scale tool and AHRQ assessment tool. [file Table3.docx]

**Table S3.** Study quality assessment using the Newcastle-Ottawa scale tool and AHRQ assessment tool

| **Study** | **Selection** | | | | **Comparability** | **Outcome** | | | **Total Stars** |
| --- | --- | --- | --- | --- | --- | --- | --- | --- | --- |
|  | **Representativeness of exposed cohort** | **Selection of non-exposed cohort** | **Ascertainment of exposure** | **Outcome of interest not present at start of study** | **Comparability of cohorts on the basis of the design or analysis** | **Assessment of outcome** | **Follow-up long enough for outcomes to occur** | **Adequacy of follow-up of cohorts** |  |
| **JONATHAN R. STEINHART-1997** | ★ | ★ | ★ | ★ | ★ | ★ | ★ | ★ | 8 |
| Siri L-1998 | ★ | ★ | ★ | ★ | ★ | ★ | ★ | ★ | 8 |
| NAM H. CHO1-2005 | ★ | ★ | ★ | ★ | ★ | ★ | ★ | ★ | 8 |
| N. Wah Cheung-2005 | ★ | ★ | ★ | ★ | - | ★ | ★ | - | 6 |
| NAM H. CHO2-2005 | ★ | ★ | ★ | ★ | ★ | ★ | ★ | - | 7 |
| Anny H Xiang-2006 | ★ | ★ | ★ | ★ | - | ★ | ★ | ★ | 7 |
| Kristian Lo¨bner-2006 | ★ | ★ | ★ | ★ | - | ★ | ★ | ★ | 7 |
| Anna J Lee-2007 | ★ | ★ | ★ | ★ | - | ★ | ★ | ★ | 7 |
| C Russell-2007 | ★ | ★ | ★ | ★ | ★ | ★ | ★ | - | 7 |
| Christian S. Go¨bl-2011 | ★ | ★ | ★ | ★ | ★ | ★ | ★ | - | 7 |
| A. H. Xiang-2011 | ★ | ★ | ★ | ★ | - | ★ | ★ | ★ | 7 |
| Tobias DK-2012 | ★ | ★ | ★ | ★ | ★ | ★ | ★ | ★ | 8 |
| Yujie Wang-2012 | ★ | ★ | ★ | ★ | - | ★ | ★ | - | 6 |
| Denice S. Feig-2013 | ★ | ★ | ★ | ★ | ★ | ★ | ★ | ★ | 8 |
| Bao W-2014 | ★ | ★ | ★ | ★ | ★ | ★ | ★ | ★ | 8 |
| Huikun Liu-2014 | ★ | ★ | ★ | ★ | ★ | ★ | ★ | ★ | 8 |
| R. Retnakaran-2015 | ★ | ★ | ★ | ★ | - | ★ | ★ | - | 6 |
| Claire E Eades-2015 | ★ | ★ | ★ | ★ | - | ★ | ★ | - | 6 |
| Valizadeh M-2015 | ★ | ★ | ★ | ★ | - | ★ | ★ | ★ | 7 |
| Joon Ho Moon-2015 | ★ | ★ | ★ | ★ | ★ | ★ | ★ | - | 7 |
| Pei-Chao LIN-2015 | ★ | ★ | ★ | ★ | - | - | ★ | - | 5 |
| Piotr Molęda-2016 | ★ | ★ | ★ | ★ | - | ★ | ★ | - | 6 |
| Catherine R Chamberlain-2016 | ★ | ★ | ★ | ★ | ★ | ★ | ★ | ★ | 8 |
| Bao W1-2016 | ★ | ★ | ★ | ★ | ★ | ★ | ★ | ★ | 8 |
| Bao W2-2016 | ★ | ★ | ★ | ★ | ★ | ★ | ★ | ★ | 8 |
| Bao W3-2016 | ★ | ★ | ★ | ★ | ★ | ★ | ★ | ★ | 8 |
| Deirdre K-2018 | ★ | ★ | ★ | ★ | ★ | ★ | ★ | ★ | 8 |
| Yukari Kugishima-2018 | ★ | ★ | ★ | ★ | - | ★ | ★ | - | 6 |
| Judith Bernstein-2019 | ★ | ★ | ★ | ★ | - | ★ | ★ | - | 6 |
| Ley SH-2020 | ★ | ★ | ★ | ★ | ★ | ★ | ★ | ★ | 8 |
| Kawasaki M-2020 | ★ | ★ | ★ | ★ | - | ★ | ★ | - | 6 |
| Dayeon Shin-2021 | ★ | ★ | ★ | ★ | - | ★ | ★ | - | 6 |
| Stefanie N Hinkle-2021 | ★ | ★ | ★ | ★ | ★ | ★ | ★ | ★ | 8 |
| Chiou YL-2021 | ★ | ★ | ★ | ★ | - | ★ | ★ | ★ | 7 |
| Anna J Wood-2021 | ★ | ★ | ★ | ★ | - | ★ | ★ | ★ | 7 |
| Enav Yefet-2022 | ★ | ★ | ★ | ★ | ★ | ★ | ★ | - | 7 |
| Jiaxi Yang-2022 | ★ | ★ | ★ | ★ | ★ | ★ | ★ | ★ | 8 |
| Mi Jin Choi-2022 | ★ | ★ | ★ | ★ | - | ★ | ★ | - | 6 |
| Roosa P-2022 | ★ | ★ | ★ | ★ | ★ | ★ | ★ | - | 7 |
| Yumei Wei-2022 | ★ | ★ | ★ | ★ | ★ | ★ | ★ | - | 7 |
| Shih-Chun Pan-2023 | ★ | ★ | ★ | ★ | ★ | ★ | ★ | ★ | 8 |
| Amir Naeh | ★ | ★ | ★ | ★ | - | ★ | ★ | - | 6 |
| Deirdre K Tobias-2024 | ★ | ★ | ★ | ★ | ★ | ★ | ★ | ★ | 8 |

| **Questions** | **Study** | | | |
| --- | --- | --- | --- | --- |
|  | Anny H. Xiang-2010 | Casagrande SS-2018 | Tawanda Chivese-2019 | Pandora L. Wander-2021 |
| **Source of Information Defined**:  Did the study clearly define the source of information (e.g., survey, record review)?  Yes or No or Unclear | Yes | Yes | Yes | Yes |
| **Inclusion and Exclusion Criteria**:  Were the inclusion and exclusion criteria for exposed and unexposed subjects (cases and controls, if applicable) listed or referenced to previous publications?  Yes or No or Unclear | Yes | Yes | Yes | Yes |
| **Time Period for Identifying Patients**:  Was the time period used for identifying patients clearly indicated?  Yes or No or Unclear | Yes | Yes | Yes | Yes |
| **Consecutiveness of Subjects**:  If not population-based, were the subjects consecutive (i.e., did the study include all patients within a specified time period)?  Yes or No or Unclear | Yes | Yes | Yes | Yes |
| **Masking of Evaluators**:  Were evaluators of subjective components of the study masked to other aspects of the participants' status?  Yes or No or Unclear | No | No | No | No |
| **Quality Assurance Assessments**:  Were any assessments undertaken for quality assurance purposes (e.g., test/retest of primary outcome measurements)?  Yes or No or Unclear | Yes | Yes | Yes | Yes |
| **Explanation of Patient Exclusions**:  Was any explanation provided for excluding patients from the analysis?  Yes or No or Unclear | Yes | Yes | Yes | Yes |
| **Assessment and Control of Confounding**:  Were measures described for assessing and/or controlling for confounding factors?  Yes or No or Unclear | Yes | Yes | Yes | Yes |
| **Handling of Missing Data**:  If applicable, was there an explanation for how missing data were handled in the analysis?  Yes or No or Unclear | Yes | Yes | Yes | Yes |
| **Response Rates and Data Completeness**:  Were the patient response rates and completeness of data collection summarized?  Yes or No or Unclear | Yes | Yes | Yes | Yes |
| **Follow-Up (if Applicable)**:  If follow-up was expected, was the percentage of patients with incomplete data or follow-up results provided?  Yes or No or Unclear | Yes | Yes | Yes | Yes |
